# Epigenetic memory of the first cell fate decision prevents complete ES cell reprogramming into trophoblast

**DOI:** 10.1038/ncomms6538

**Published:** 2014-11-26

**Authors:** Francesco Cambuli, Alexander Murray, Wendy Dean, Dominika Dudzinska, Felix Krueger, Simon Andrews, Claire E. Senner, Simon J. Cook, Myriam Hemberger

**Affiliations:** 1 Epigenetics Programme, The Babraham Institute, Cambridge CB22 3AT, UK; 2 Centre for Trophoblast Research, University of Cambridge, Downing Street, Cambridge CB2 3EG, UK; 3 Bioinformatics Group, The Babraham Institute, Cambridge CB22 3AT, UK; 4 Signalling Programme, The Babraham Institute, Cambridge CB22 3AT, UK; 5 Wellcome Trust—Medical Research Council Cambridge Stem Cell Institute, Tennis Court Road, Cambridge CB2 1QR, UK

## Abstract

Embryonic (ES) and trophoblast (TS) stem cells reflect the first, irrevocable cell fate decision in development that is reinforced by distinct epigenetic lineage barriers. Nonetheless, ES cells can seemingly acquire TS-like characteristics upon manipulation of lineage-determining transcription factors or activation of the extracellular signal-regulated kinase 1/2 (Erk1/2) pathway. Here we have interrogated the progression of reprogramming in ES cell models with regulatable *Oct4* and *Cdx2* transgenes or conditional Erk1/2 activation. Although trans-differentiation into TS-like cells is initiated, lineage conversion remains incomplete in all models, underpinned by the failure to demethylate a small group of TS cell genes. Forced expression of these non-reprogrammed genes improves trans-differentiation efficiency, but still fails to confer a stable TS cell phenotype. Thus, even ES cells in ground-state pluripotency cannot fully overcome the boundaries that separate the first cell lineages but retain an epigenetic memory of their ES cell origin.

## Abstract (web summary)

Various strategies have been reported to generate mouse trophoblast stem cells (TSCs) from mouse embryonic stem cells (ESCs). Here the authors show that, regardless of the strategy used for TSC generation, these retain an epigenetic and transcriptional memory of the ESC origin and the transition remains incomplete.

## Graphical abstract

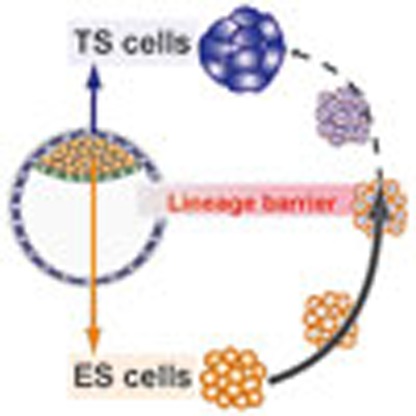

Cell fate specification is achieved through a close interplay between signalling pathways and transcription factors, leading to a progressive restriction of cellular plasticity that ultimately results in terminal differentiation^1^,^2^,^3^. These differentiation events are accompanied by the acquisition of cell lineage- and cell type-defining epigenetic landscapes that lock in the acquired fate and normally prevent de-differentiation^2^,^4^. Reprogramming aimed at reverting the developmental potential of somatic cells back to pluripotency has been achieved by a combination of only four transcription factors that are able to largely overcome the established epigenetic barriers and reset cellular plasticity to a state akin to that of embryonic stem (ES) cells^5^.

A strategy that may prove even more powerful than iPS cell reprogramming in the therapeutic context is that of direct trans-differentiation of one somatic cell type into another^6^,^7^. Remarkably, insights from these approaches have provided strong support for the validity of Waddington’s concept of the canalization of developmental pathways, which predicts that the more closely related two cell types are developmentally, the easier it is to overcome the separating barriers in reprogramming strategies.

Our interest is in the first differentiation event after fertilization in which cells of the extraembryonic trophoblast lineage are irrevocably set apart from cells that will go on to form the embryo proper^8^. This event becomes manifest at the blastocyst stage with the formation of the trophectoderm (TE) and the inner cell mass (ICM), and later epiblast, that establish the trophoblast and embryonic cell lineages, respectively. Numerous elegant embryological and genetic studies have unequivocally shown that by the late-blastocyst stage, commitment to these cell lineages is irreversibly fixed such that TE cells exclusively contribute to extraembryonic trophoblast cell types of the yolk sac and placenta, whereas all somatic cell types of the embryo proper, as well as the germ line, descend from the ICM/epiblast^9^,^10^. This strict cell fate commitment is retained in stem cells that can be derived from the mouse blastocyst. Thus, ES cells derived from the ICM/epiblast are pluripotent with the capacity to differentiate into all somatic cell types of the adult but are generally excluded from differentiating into trophoblast derivatives; conversely, trophoblast stem (TS) cells derived from the TE are committed to a trophoblast cell fate^11^,^12^,^13^. At the epigenetic level, commitment to the first cell lineages is reinforced by the establishment of unique DNA methylation profiles, which ensure the restriction of cell fate during future development^14^,^15^. In line with their retained cell lineage restrictions, ES and TS cells are unambiguously defined by distinct DNA methylomes, which dictate their developmental plasticity and differentiation trajectories^16^.

Although the first differentiation event is considered irreversible in normal conditions, trans-differentiation between the embryonic and trophoblast lineages has been reported to occur in distinct experimental settings. Thus, in line with their role in driving cell fate decisions during development, episomal expression of the early trophoblast transcription factors Tead4, Cdx2, Eomes, Tcfap2c, Gata3 and Elf5, or downregulation of the pluripotency factor Oct4 (encoded by the *Pou5f1* gene), can induce trophoblast cell fate in ES cells^15^,^17^,^18^,^19^,^20^,^21^. Conversely, TS cells can be reprogrammed to ES-like cells by forced expression of the ‘Yamanaka’ factors, although at reduced efficiency compared with somatic cells^22^. Although overexpression of specific transcription factors is commonly regarded as the key initiator of cellular reprogramming, these strategies also depend on the extracellular environment provided by the culture medium, which activates or inhibits signalling pathways to support the reprogramming process^23^,^24^. Remarkably, in the context of ES-to-TS cell reprogramming, constitutive activation of the H-Ras GTPase, a molecular switch that activates the extracellular signal-regulated kinase 1/2 (Erk1/2) signalling cascade, was reportedly sufficient to convert ES into TS-like cells by strongly activating Cdx2 (ref. 25). This finding suggested that extracellular signals may directly govern cell fate decisions and be sufficient to induce conversions between established cell lineages.

Studying ES-to-TS cell reprogramming holds great promise for deciphering the molecular processes that initiate the first cell fate specification event in early development and for identifying the cues that target DNA methylation and other epigenetic modifications to distinct loci in a cell lineage- and cell type-specific pattern. Here we interrogated models of transcription factor modulation (iCdx2, Oct4-cKO) and Ras-Erk1/2 activation (iRas, iRaf) for the progression of ES-to-TS cell conversion, with the aim of unravelling the dynamics of epigenomic reprogramming events required to underpin this process. We show that, despite the developmental plasticity of ES cells, the TS-like cells obtained from these models exhibit important differences when compared with ‘genuine’ TS cells (derived from normal fertilized embryos) and retain a distinct epigenetic memory of their ES cell precursors. We identify a core set of differentially methylated lineage ‘hallmark’ loci that remain incompletely reprogrammed and normally safeguard cells from trans-differentiation between the embryonic and trophoblast lineages. Together, these core genes provide an unambiguous lineage signature and serve as unique identifier of early lineage origin. Their epigenetic regulation is central to enforcing the first cell fate decision in development and to dictating the developmental potency of early embryo-derived stem cells.

## Results

### Establishment of inducible Erk1/2 ES cell models

**Figure 1:**
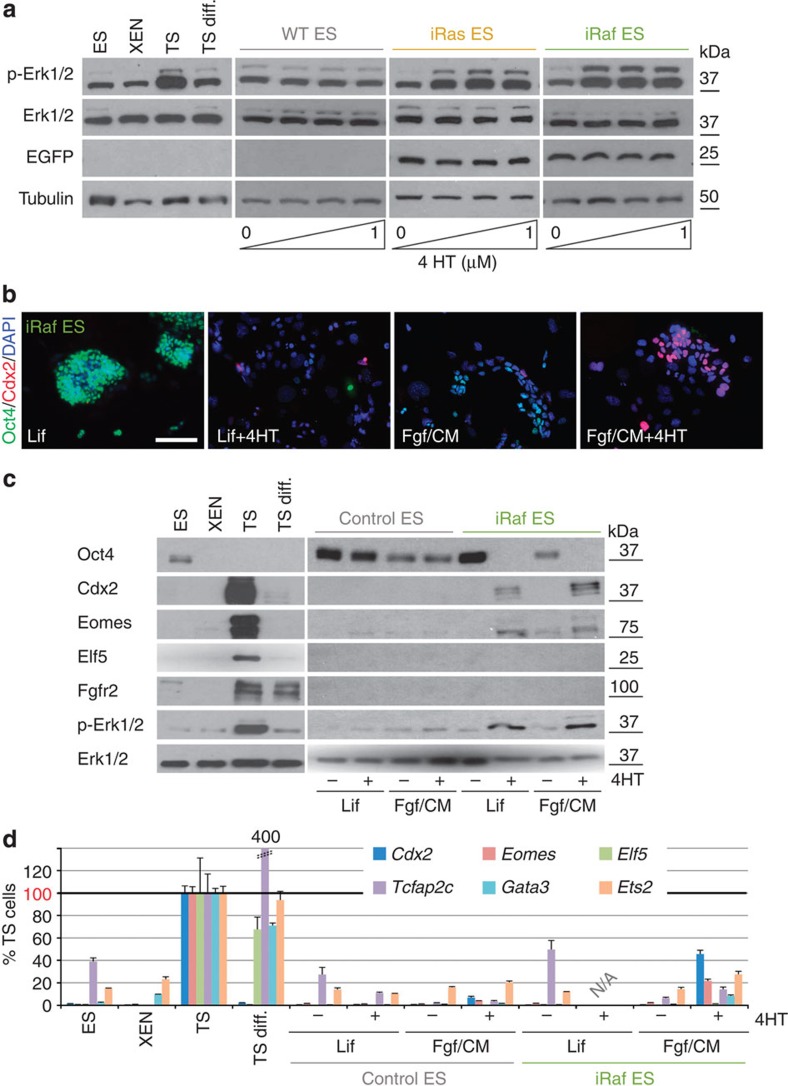
Constitutive Ras and Raf activation in ES cells leads to Cdx2 upregulation but is insufficient to induce trophoblast differentiation. (**a**) Established iRas and iRaf ES cells, as well as wild-type (WT) control ES cells, were cultured in TS cell conditions for 24 h in the presence of increasing concentrations of 4HT. Erk1/2 phosphorylation at levels similar to those in TS cells was achieved in the chosen ES cell clones. (**b**) Immunofluorescence staining for Oct4 and Cdx2 of iRaf ES cells after 7 days of culture in ES cell conditions (‘Lif’) or in TS cell conditions (‘Fgf/CM’) in the presence or absence of 4HT. Raf kinase activation in TS cell conditions induced high levels of Cdx2 but these cells did not necessarily acquire trophoblast morphology. Scale bar: 100 μm. (**c**) Western blot of iRaf and control ES cells (stably transfected with an empty EGFP expression construct) after 6 days of culture in the indicated conditions. TS cell conditions in the presence of 4HT induced Cdx2 and Eomes, albeit not to TS cell levels. Elf5 and Fgfr2 were not induced, even upon long exposure (Supplementary Fig. 8b). (**d**) RT–qPCR analysis of control and iRaf ES cells after 7 days of culture in the indicated conditions, normalized to TS cell values and displayed as mean±s.e.m. of three biological replicates. *Cdx2* was activated by Raf induction in TS cell conditions to ~50% TS cell levels; other TS cell-characteristic transcription factors were expressed at levels far lower than those in TS cells.

To study the progression and fidelity of mouse ES cell trans-differentiation towards the trophoblast lineage, we first focussed on signalling pathways because of their function upstream of transcription factors. Prompted by the report that a constitutively active *H-Ras* GTPase allele can cause ES cells to adopt TS cell-like (TSL) fate, we generated ES cell lines stably expressing inducible versions of Ras (iRas) and Raf-1 (iRaf), an effector kinase downstream of Ras that specifically activates the Mek1/2-Erk1/2 signalling pathway^26^,^27^. Both iRas and iRaf constructs allowed conditional, 4-hydroxytamoxifen (4HT)-dependent activation of Erk1/2, assessed by Erk1/2 phosphorylation (p-Erk1/2) in stable ES and HEK293 cells (Fig. 1a and Supplementary Fig. 1a,b). iRas and iRaf ES cell clones were selected that exhibited levels of 4HT-dependent p-Erk1/2 comparable to that observed in TS cells (Fig. 1a), and the specificity of signal cascade activation was confirmed (Supplementary Fig. 1c,d).

### Erk1/2-induced Cdx2 is insufficient for TSL reprogramming

Differentiation towards a TSL fate was first investigated using the iRaf ES cells, upon culture in TS cell conditions with 4HT for 6–7 days. As expected, under these conditions the majority of cell colonies acquired a flat epithelial-like morphology reminiscent of TS cells. Raf activation, both under ES and TS cell conditions, was associated with a sharp downregulation of Oct4 in the entire cell population and a concomitant increase in Cdx2 and Eomes (Fig. 1b,c). However, high Cdx2 expression levels in individual cells did not correlate with the acquisition of TSL characteristics, and these cells did not form TSL colonies. Global Cdx2 and Eomes protein levels remained well below that characteristic of TS cells. Other critical TS cell factors, notably Elf5 and Fgfr2c, remained undetectable (Fig. 1c). We also assessed these cells for mRNA expression levels of a series of TS cell transcription factors. It is important to note that, while these transcription factors have key roles in trophoblast development, they are not exclusive to the trophoblast lineage and many of them, such as *Cdx2*, *Eomes, Tcfap2c* and *Ets2*, become upregulated in the embryo proper soon after implantation *in vivo* and upon ES cell differentiation *in vitro*^28^,^29^,^30^,^31^,^32^,^33^. Setting the stringent measure of expression levels in ‘genuine’ TS cells as 100%, iRaf ES cells failed to upregulate any of these genes above 50% of TS levels even when the trans-differentiation period was extended to 15 days (Fig. 1d and Supplementary Fig. 2a). Addition of Bmp4 to iRaf cells led to an increase in *Cdx2* expression levels, yet still failed to induce other trophoblast-characteristic transcription factors to TS-equivalent levels (Supplementary Fig. 2a).

Thus, *Cdx2* appears to be a downstream transcriptional target of Raf-Erk1/2 activation (either direct or indirect), yet its upregulation *per se* is not sufficient to induce TSL trans-differentiation in iRaf ES cells.

### Comparative analysis of ES-to-TSL reprogramming progression

**Figure 2:**
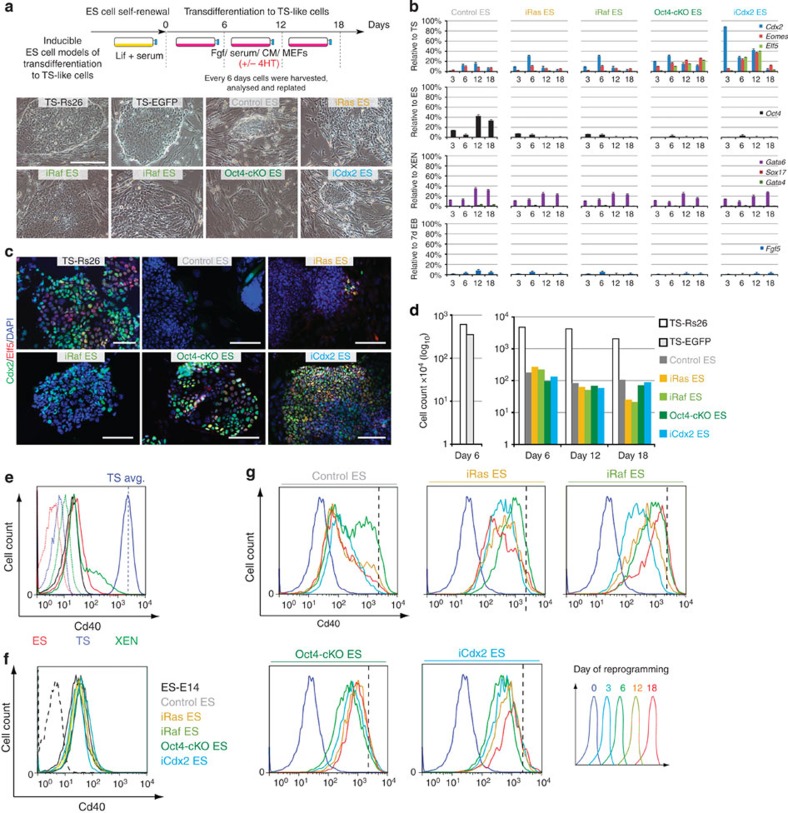
Comparative analysis of ES-to-trophoblast stem cell-like (TSL) trans-differentiation models. (**a**) Schematic representation of the experimental set-up of the trans-differentiation time course analysis and phase contrast images of representative cell colonies 18 days after the induction of trophoblast differentiation. Scale bar: 100 μm. (**b**) mRNA expression levels of the core trophoblast transcription factors *Cdx2*, *Eomes* and *Elf5*, as well as other embryonic lineage markers, in the TSL models throughout the trans-differentiation time course. Transcript abundance remained unstable for all three genes across stages. No differentiation into primitive endoderm (*Gata4*, *Gata6* and *Sox17*) or epiblast (*Fgf5*) derivatives was observed. Data are displayed as mean±s.e.m. of three biological replicates. EB, embryoid body. (**c**) Immunofluorescence staining for Cdx2 and Elf5 proteins in TSL models after 12 days of reprogramming. Cdx2 expression was observed in all models but did not coincide with Elf5 expression or the adoption of TS cell morphology on an individual cell level. Significant numbers of Elf5-positive cells were only observed in Oct4-cKO and iCdx2 TSL models. Scale bars: 100 μm. (**d**) Quantification of the proliferation rates in TSL models, that remained much reduced at 1.5 orders of magnitude below those of two independent *bona fide* TS cell lines. (**e**) FACS analysis using Cd40 as TS cell-specific cell surface marker not (or not significantly) expressed in ES and XEN cells. Dotted lines are secondary antibody-only controls. The average TS cell intensity value is indicated by the vertical dashed line. (**f**) ES cell lines used for the induction of trophoblast differentiation were Cd40^low^ when grown in standard ES cell conditions, indistinguishable from wild-type control ES cells. (**g**) Flow cytometric analysis of the progression of ES-to-TSL differentiation after 3, 6, 12 and 18 days. Control ES cells transiently upregulated Cd40 on day 6 as differentiation into epiblast-like cells takes place, but subsequently reverted back to Cd40^low^ levels. iRas and iRaf ES cells upregulated Cd40 but remained very heterogeneous. Oct4-cKO and iCdx2 cells upregulated Cd40 homogenously population-wide, but importantly, never acquired the Cd40^high^ levels characteristic of genuine TS cells, represented by the dotted vertical line. Data are of three biological replicates.

To compare the efficiency of TSL differentiation in our signalling activation models to that upon direct transcription factor modulation, we included ES cell lines with tetracycline-/doxycycline-regulatable *Oct4* repression (Oct4-cKO, previously termed Zhbtc4 (ref. 21)) as well as with inducible *Cdx2* overexpression (iCdx2), established using a previously validated Cdx2:ER-fusion construct^18^, in our reprogramming approaches. Together with control enhanced green fluorescent protein (EGFP)-transfected ES and normal TS cells, all TSL models were subjected in parallel to a time course experiment over 18 days, a time frame designed to cover the 4- to 8-day period necessary for TSL transition according to previous reports^18^,^25^,^34^. In keeping with our expectations, pluripotency features were rapidly lost (Supplementary Fig. 2b) and some TS-like characteristics acquired in all models. iRas ES cells exhibited the least efficient TSL differentiation. Often, they rather displayed morphological characteristics of ES cells undergoing somatic differentiation with fibroblast-like cells emanating at the periphery of colonies, while iRaf cells exhibited a mixed phenotype with only some colonies presenting TS-like features (Fig. 2a). Although *Oct4* was downregulated, Cdx2 expression was patchy and Elf5-positive cells extremely rare in both these models (Fig. 2b,c). By comparison, Oct4-cKO and iCdx2 cells changed very homogeneously into flat, epithelial-like colonies with continuous boundaries typical of TS cells (Fig. 2a). They robustly downregulated *Oct4* and exhibited widespread expression of the trophoblast transcription factors Cdx2 and Elf5, while no markers of other lineages were upregulated (Fig. 2b,c). The induction of Elf5 is of particular note as the *Elf5* promoter is methylated and repressed in ES cells, and must be epigenetically reprogrammed to a hypomethylated state for transcription to occur^15^. Nevertheless, all TSL cell models could be unequivocally distinguished from bona fide TS cells by cell and colony size, as they exhibited more than 10-fold reduced proliferation rates compared with TS cells (Fig. 2d). In addition, the abundance of TS cell transcription factors fluctuated significantly between time points within each line, and did not reach consistent, stable TS cell-equivalent levels at any stage (Fig. 2b).

The recent identification of lineage-specific cell surface markers^35^ allowed us to monitor the progression of ES-to-TSL reprogramming in individual cells on a population-wide scale across the time course (Fig. 2e). As expected, under ES cell conditions all models were indistinguishable from wild-type control ES cells with very low levels of the TS cell-enriched surface marker Cd40 (Cd40^low^) compared with TS cells (Cd40^high^; Fig. 2f). Upon culture under TS cell conditions, control ES cells upregulated Cd40 transiently, consistent with expression of this antigen during differentiation towards early epiblast cells^35^. In contrast, all TSL models shifted towards Cd40^medium^ levels as early as 3 days after the induction of trans-differentiation, with relatively little further increase during the subsequent 15 days of the experiment (Fig. 2g). Oct4-cKO and iCdx2 TSL cells exhibited a sharper Cd40 peak than iRas and iRaf cells indicative of a more homogenous phenotype, in line with their morphological appearance. However, even in these models, Cd40 never reached TS cell levels. We also tested ES cells with combined overexpression of *Cdx2* and Raf activation, but found that this did not significantly enhance Cd40 surface expression (Supplementary Fig. 3a–c), thus supporting our previous notion that Erk1/2 signalling acts predominantly upstream of Cdx2 in this context.

Taken together, these data indicate that differentiation towards the trophoblast lineage occurs in all reprogramming models but is more efficient and uniform upon modulation of lineage-specifying transcription factors, compared with the Ras-Raf-Erk1/2 signalling pathway. Importantly though, this reprogramming process stalls before acquiring a full TS cell phenotype in all models tested.

### Epigenomic reprogramming

**Figure 3:**
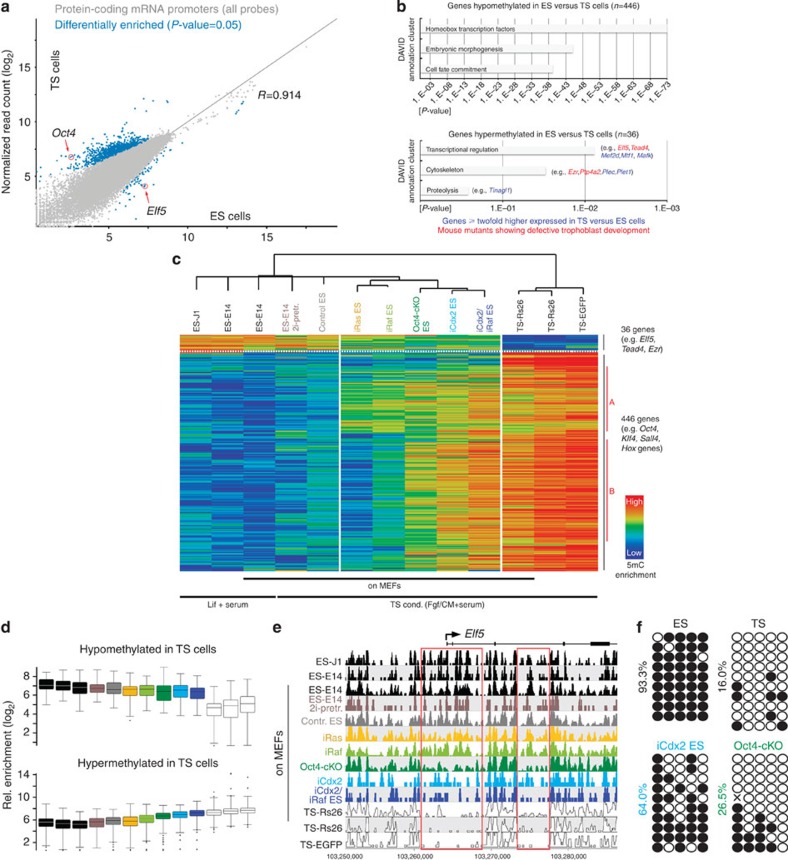
Epigenomic reprogramming dynamics of DNA methylation in TSL models. (**a**) Scatter plot of meDIP-seq replica sets of two independent ES and TS cell lines each identified 482 signature loci that are differentially methylated between the two stem cell types. Each dot represents a 5-kb probe located in a 10-kb region centred on transcription start sites (using three overlapping 5 kb probes with 2.5 kb step size). Blue dots correspond to differentially enriched probes. (**b**) Functional annotation and classification of genes identified in (**a**) that are either hypo- or hypermethylated in ES versus TS cells. In red are genes whose mouse mutants show defective trophoblast development according to the MGI database. In blue are genes found to be more than twofold higher expressed in TS versus ES cells^37^. (**c**) Heatmap and neighbour joining tree based on a Pearson’s correlation distance matrix of the DNA methylation levels at the 482 signature loci. Subcluster ‘A’ identifies genes that gain methylation in all reprogramming models, including upon Ras/Raf activation, whereas subcluster ‘B’ identifies loci that become significantly reprogrammed only upon modulation of transcription factors. (**d**) Box whisker plots of the progression of methylation reprogramming in the various TSL models, compared with ES and TS cells. This representation visualizes that the major roadblock to reprogramming is the failure to demethylate trophoblast-specific ‘gatekeeper’ genes. In comparison, the gain of methylation is generally more comprehensively achieved. (**e**) Distribution of DNA methylation at the *Elf5* locus in all TSL models. Red boxes indicate important regulatory regions where this lack of complete demethylation reprogramming is exemplified. (**f**) Bisulphite-sequencing profiles of the *Elf5* promoter; the observed DNA methylation levels were in agreement with those determined by meDIP-seq.

As ES and TS cells exhibit distinct and globally divergent DNA methylation profiles^16^, transitioning between these two stem cell types requires profound epigenetic reprogramming events to occur. To unravel the dynamics of this process, we determined the DNA methylation profiles by 5-methylcytosine (5mC) immunoprecipitation coupled to high-throughput sequencing (meDIP-seq) on all ES-to-TSL reprogramming models in comparison with bona fide ES and TS cells. To get a broad overview of methylation differences at relevant genomic sites, we first compared 10 kb regions centred around transcriptional start sites between two independent ES and TS cell lines each. This identified 482 loci that exhibited a highly stem cell type-specific methylation pattern (Fig. 3a). In line with our previous data^16^, gene promoters hypermethylated in ES compared with TS cells were comparatively rare, with only 36 loci identified using the current probe definition, including the particularly stringently regulated gatekeeper gene *Elf5* (Fig. 3b). In contrast, many more promoters exhibited higher methylation enrichment in TS cells compared with ES cells, notably at genes involved in embryonic morphogenesis and patterning. This group of 482 differentially methylated loci represents an epigenetic signature of stem cell identity and therefore was used to assess the extent of ES-to-TSL trans-differentiation. Although a shift towards a more TS-like epigenotype was observed in all TSL models, their methylation profiles remained at an intermediate stage in between the boundaries set by ES and TS cells (Fig. 3c). Importantly, a closest-neighbour clustering analysis of the 482 signature loci clearly separated bona fide TS cells from all TSL models that remained epigenomically more closely related to their ES cell origin. Overall, *de novo* methylation of embryo-specific genes was more frequently achieved than demethylation of trophoblast-specific loci (Fig. 3c,d). Among the loci that need to gain methylation in the ES-to-TS cell transition, we identified two subclusters of genes, termed ‘A’ and ‘B’, that either acquired methylation in all TSL models or preferentially only in the transcription factor modulation models, respectively. A small set of genes was also observed that failed to gain methylation in all models. Overall, however, the major barrier to reprogramming into TS-equivalent cells was imposed by differentially methylated regions (DMRs) that are highly methylated in ES cells and hypomethylated in TS cells (‘ES-DMRs’) and thus need to lose methylation in TSL models to reflect the trophoblast epigenotype (Fig. 3c,d). These regions, on the whole, failed to fully reprogramme, as exemplified by the *Elf5* locus that exhibited a gradual, stochastic loss of methylation in the various TSL models but never reached the very hypomethylated state characteristic of TS cells (Fig. 3e,f and Supplementary Fig. 3d).

### ES cell memory is resistant to 2i-induced hypomethylation

**Figure 4:**
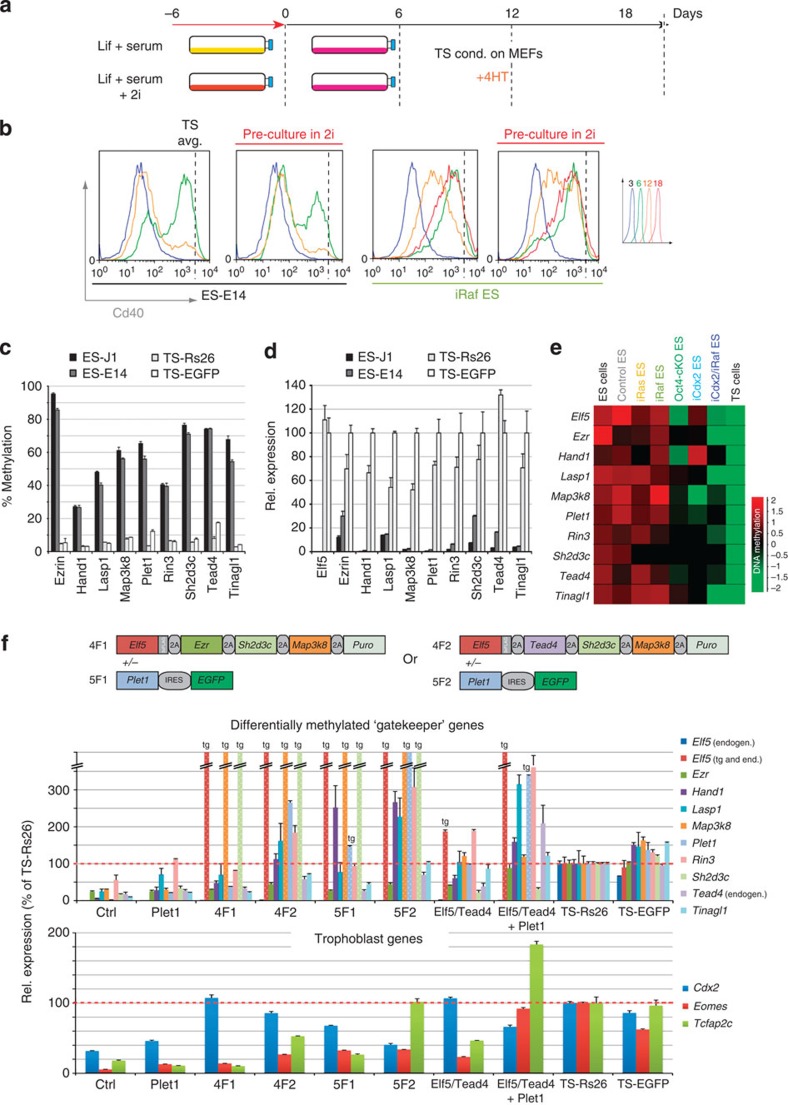
A persistent epigenetic memory at differentially methylated lineage gatekeeper genes. (**a**) Layout of the experimental protocol of pre-treatment of ES cells with Mek1/2 and Gsk3 inhibitors (2i) that push ES cells into the ground-state of pluripotency and induce global hypomethylation. (**b**) Time course of the acquisition of Cd40 surface antigen expression as in Fig. 2g revealed no further improvement to reprogramming. Data are of three biological replicates. (**c**) Sequenom analysis confirmation of the differential methylation status of nine genes identified by meDIP-seq (Fig. 3) that are, like *Elf5*, highly methylated in two independent ES cell lines but hypomethylated in TS cells. (**d**) RT–qPCR verifying the differential expression of these genes, with significantly higher expression levels observed in TS cells, as expected. Data are displayed as mean±s.e.m. of three replicates. (**e**) Heatmap of methylation reprogramming at these loci, focussed on the differentially methylated regions assessed in (**a**), across all TSL models. No single locus completely escaped reprogramming entirely, but also no single TSL model induced the most profound demethylation across all loci. (**f**) Generation of co-expression constructs driving concomitant expression of four to five gatekeeper genes as shown, or *Elf5*-*Tead4-Puromycin* (‘Elf5/Tead4’) separated by 2A sites only. E14 ES cells were (co-)transfected with these constructs and then cultured in TS cell conditions for 8 days. Expression levels of gatekeeper genes, as well as of the three important TS cell transcription factors *Cdx2*, *Eomes* and *Tcfap2c*, were determined by RT–qPCR. Values are expressed relative to TS-Rs26 cells as mean±s.e.m.

Given that the major hurdle to complete reprogramming was imposed by ES-DMRs, we asked whether culture of ES cells in ‘2i’ conditions, that is, in the presence of Mek1/2 and Gsk3 inhibitors that induce global hypomethylation and promote the naïve state of pluripotency^23^,^24^,^36^, could enhance trans-differentiation efficiency (Fig. 4a). As expected, control experiments showed that 2i exposure indeed caused a profound loss of methylation at ES-DMRs, and that a globally hypomethylated background (as observed in *Dnmt1*-deficient ES cells) resulted in the activation of many of the ES-DMR-associated ‘gatekeeper’ genes (Supplementary Fig. 4a,b). Thus, we tested the effect of 2i exposure before the start of trans-differentiation on wild-type and iRaf ES cells, but did not find an improvement in the efficiency or progression of reprogramming upon this pre-treatment (Fig. 4b). The lack of enhanced reprogramming was reflected by a virtually unchanged methylation pattern at the differentially methylated signature loci (Fig. 3c–e), presumably because of rapid re-methylation of the genome in an embryonic lineage-specific pattern upon transfer of cells into the serum-containing TS cell culture conditions.

### Lineage ‘gatekeepers’ underpin the first lineage decision

Since failure to demethylate ES-DMRs of trophoblast-specific genes appeared to constitute a major impediment to complete reprogramming, we aimed to identify functionally relevant genes that are, similar to *Elf5*, methylated and repressed in ES cells but unmethylated and expressed in TS cells. Therefore, we first defined a core set of trophoblast-specific genes based on differential expression in ES and TS cells^37^. Restricting our search to this subset, we analysed the meDIP-seq data for gene promoters, using a tighter probe definition of 1 and 2 kb probes upstream of transcriptional start sites, that exhibited at least fourfold read count differences between the ES and TS cell lines. This procedure led us to select nine loci in addition to *Elf5*, including the key trophoblast transcription factors Tead4 and Hand1 (refs 17, 38), that were robustly methylated in ES cells but hypomethylated in TS cells, and conversely expressed predominantly in TS cells (Fig. 4c,d). All 10 loci underwent some degree of methylation reprogramming in the TSL models after 12 days of trans-differentiation, yet failed to reach the degree of hypomethylation characteristic of TS cells and accordingly did not become activated to TS cell expression levels (Fig. 4e and Supplementary Fig. 4c).

With the rationale that forced expression of these incompletely reprogrammed trophoblast genes may improve trans-differentiation efficiency, we generated several constructs that allowed concomitant co-expression of the most prominent candidates (Fig. 4f). These were selected for their known functions as part of the TS cell transcriptional network (*Elf5* and *Tead4*), in early cell fate specification and epithelial integrity (*Ezr*), expression in the TS cell niche *in vivo* (*Plet1*), upstream activators of Erk1/2 signalling (*Map3k8*) or because of their particular resistance to reprogramming and transcriptional activation in all models (*Sh2d3c*)^39^,^40^,^41^,^42^,^43^. Using wild-type ES cells to identify the most efficient factor combinations, we observed an increase in gatekeeper gene expression over ES cell levels in particular with the 4F2 (*Elf5*, *Tead4*, *Sh2d3c* and *Map3k8*) construct with and without additional *Plet1* expression (Fig. 4f). Much of this beneficial effect was observed even with *Elf5* and *Tead4* (E+T) alone. While Plet1 helped to activate some gatekeeper genes such as *Lasp1*, it triggered hyperactivation of *Hand1* and *Tcfap2c* indicative of trophoblast differentiation^44^. Endogenous *Elf5* remained hardly expressed at all (Fig. 4f).

**Figure 5:**
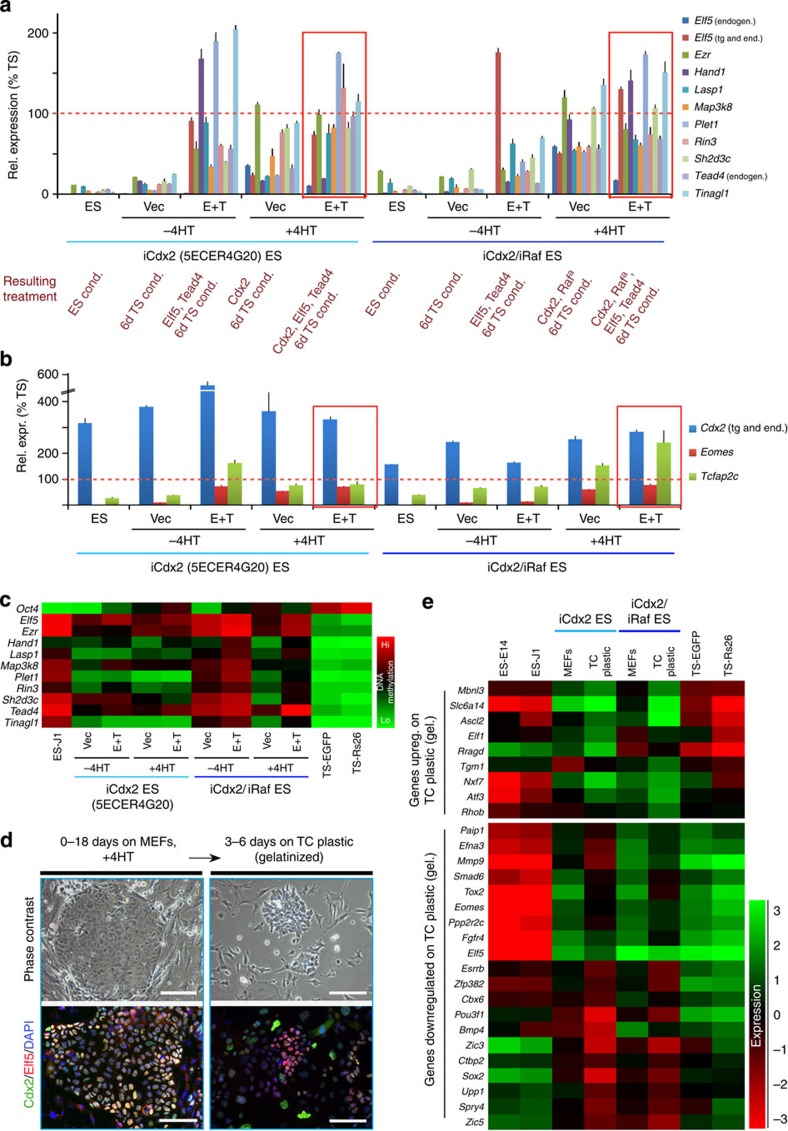
The three core TS cell transcription factors Cdx2, Elf5 and Tead4 are insufficient to complete reprogramming to induce a stable TS cell phenotype in bulk culture. (**a**) iCdx2 cells, shown previously to be capable of placental contribution^18^, and iCdx2/iRaf cells were transfected with an Elf5-2A-Tead4-2A-Hygro^R^ (E+T) or Hygro^R^-only control (vec) expression construct, subjected to the trans-differentiation protocol for 6 days on antibiotic-resistant MEFs, FACS-purified from MEFs and assessed for gatekeeper gene expression levels by RT–qPCR. Details of overexpressed genes resulting from the various conditions are given in red. *Elf5* and *Tead4* expression, in conjunction with *Cdx2* activation, improves the activation of some of the remaining gatekeeper genes. Values are of three biological replicates and expressed as percent of TS cell expression levels combined of the two independent TS cell lines (mean±s.e.m.). (**b**) Activation of TS cell transcription factors in the same cells as in (**a**). Hyperactivation of *Tcfap2c* and *Hand1* (in **a**) indicate onset of terminal trophoblast differentiation. Values expressed as in **a**. (**c**) Dynamics of methylation reprogramming of these cells at the ES-DMRs identified by meDIP-seq and assessed by Sequenom analysis as in Fig. 4c. (**d**) Assessment of phenotypic stability of reprogrammed TSL (iCdx2) cells. Upon removal of the MEF layer, the tight epithelial colony shape that is characteristic of bona fide TS cells was rapidly lost, and cells failed to maintain the co-expression of Cdx2 and Elf5 that is required to preserve the proliferative, self-renewing TS cell state, and instead started to differentiate. Scale bars: 200 μm (phase contrast), 100 μm (immunofluorescence). (**e**) RNA-seq analysis of iCdx2 (5ECER4G20) and iCdx2/iRaf ES cells grown for 5 days on MEFs, and then replated for 3 days on gelatinized TC plastic. Note the instability of gene expression, and specifically the downregulation of critical TS cell genes such as *Eomes*, *Elf5*, *Esrrb* and *Sox2* upon transfer of reprogrammed TSL cells from MEFs onto TC plastic.

We then tested whether this at least partially beneficial effect of *Elf5* and *Tead4* on some of the gatekeeper genes could enhance TSL differentiation in the most advanced reprogramming models, that is, in an iCdx2 ES cell line (‘5ECER4G20’) that had been shown to be capable of placental contribution in chimeras^18^ and in our iCdx2/iRaf cell line. When *Cdx2*, *Tead4* and *Elf5* were co-induced, most of the remaining gatekeeper genes, as well as *Eomes* and *Tcfap2c*, were upregulated (Fig. 5a,b, red boxes). However, as observed before, achieving stable expression at TS cell levels proved to be a major shortfall, such that key genes including *Eomes*, *Elf5* and *Hand1* remained well below TS cell levels in iCdx2 ES cells; while Raf activation led to an increase in their expression, this tended to hyperinduce other gatekeepers and again triggered the onset of differentiation markers (*Hand1* and *Tcfap2c*). Similarly, assessment of the epigenetic profile at the lineage signature ES-DMRs demonstrated an incomplete reprogramming in all models, with particularly refractory reprogramming at the *Elf5*, *Ezr*, *Map3k8* and *Tead4* loci (Fig. 5c). This persistent difference of TSL cells from bona fide TS cells became strikingly evident when ‘reprogrammed’ cells were plated in the absence of mouse embryonic fibroblast (MEF) feeder cells commonly used to induce trans-differentiation. Unlike bona fide TS cells, TSL cells rapidly lost the tight epithelial morphology, as well as the temporarily acquired co-expression of Cdx2 and Elf5 that is critical for TS cell self-renewal when the supportive MEF layer was removed (Fig. 5d and Supplementary Fig. 5). Global expression profiling further corroborated the labile TSL phenotype as key TS cell genes including *Esrrb*, *Eomes*, *Elf5*, *Bmp4*, *Spry4* and *Sox2* were quickly downregulated upon MEF removal (Fig. 5e). Thus, these cells had not acquired a stable TS cell phenotype.

### Dissecting cell heterogeneity in the reprogramming models

**Figure 6:**
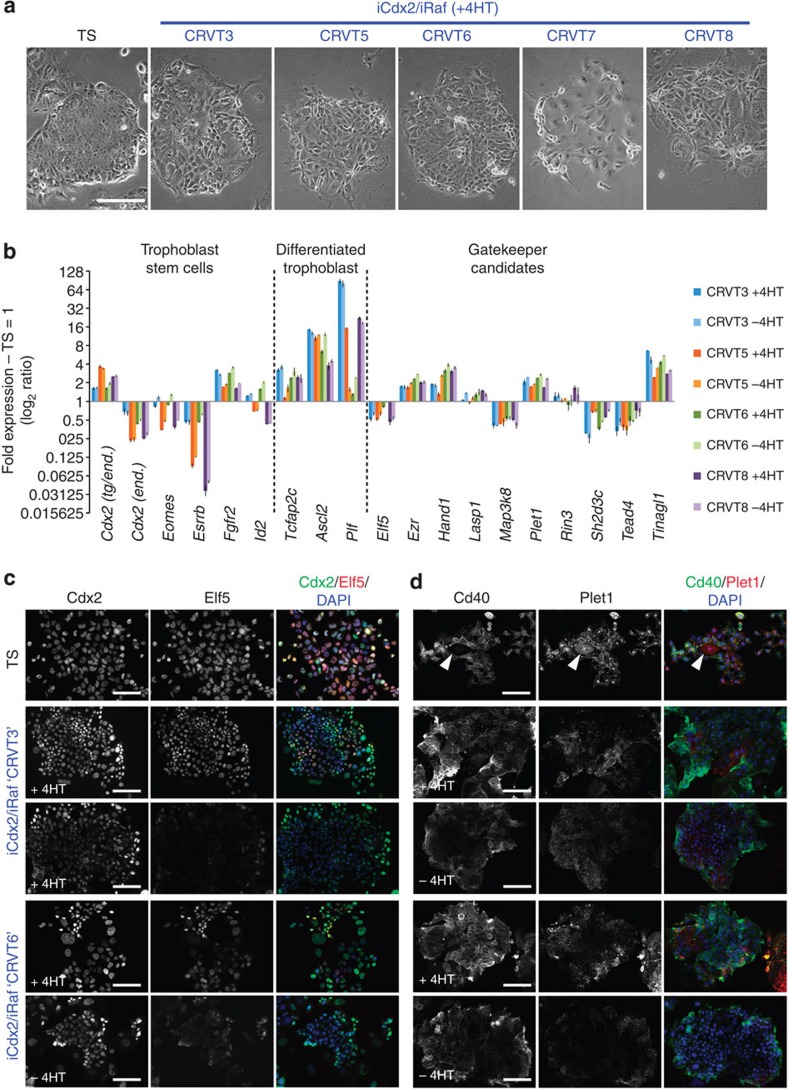
Selection strategy by epithelial morphology in the most efficient iCdx2/iRaf reprogramming model. (**a**) Phase contrast images of TSL clones selected on the basis of colony shape and epithelial morphology. After an initial 5-day reprogramming on MEFs, cells were passaged for 5 weeks on tissue culture plastic in TS cell conditions in the presence of 4HT, during which time colonies were picked and expanded. Clone heterogeneity is observed, with clones CRVT3 and CRVT6 exhibiting the most genuinely TS-like epithelial colony shape. Scale bar: 200 μm. (**b**) Expression analysis of a cohort of trophoblast stem cell and differentiation-associated genes, as well as the identified gatekeeper loci, in selected clones. After growing/expanding colonies in the presence of 4HT for 5 weeks, cells were seeded ±4HT and RNA harvested after 2 days. Tight epithelial morphology in clones CRVT3 and CRVT6 correlates with TS-like *Eomes* expression levels. Overall, however, differentiation-associated markers are vastly upregulated, and gatekeeper gene expression remains unbalanced. Lack of transcriptional activation of *Elf5*, *Map3k8*, *Sh2d3c* and *Tead4* is evident. Values are of three replicates and expressed as mean±s.e.m. (**c**) Immunostaining on these clones for Cdx2 and Elf5. Frequent lack of activation or re-silencing of Elf5, correlated with a more pronounced tendency to differentiate, is observed. (**d**) Immunostaining for TS cell surface markers Cd40 and Plet1. Plet1 is a GPI-anchored membrane-associated protein in TS cells, but is redistributed towards a cytoplasmic localization in differentiating trophoblast cells (arrowhead). Despite the TS-like colony shape, reprogrammed cell clones are extremely variable for Cd40 and largely fail to express Plet1 on their cell surface in a TS-like pattern and intensity. All photographs in **c**,**d** were taken at precisely the same exposure settings. Scale bars: 100 μm.

We then asked whether, despite the incomplete reprogramming in bulk culture, individual cells might still have acquired a genuine induced-TS (iTS) cell phenotype. To address this issue of cell heterogeneity, we selected clones based on most TS-like epithelial morphology in the overall best-performing iCdx2/iRaf cell line (Fig. 5). After initial induction of reprogramming on MEFs, cells were passaged in the presence of 4HT for a further 5 weeks, when colonies were picked, expanded and assessed in the presence and absence of 4HT to determine the stability of the Cdx2- and Raf-induced phenotype. Morphologically, a number of clones maintained a remarkably TS-like epithelial character (Fig. 6a), which coincided with *Eomes* expression reaching levels comparable to TS cells (Fig. 6b). Nonetheless, expression of gatekeeper genes and trophoblast markers exhibited major imbalances compared with bona fide TS cell lines, even after long-term culture (Fig. 6b and Supplementary Fig. 6). Again, a common failure to maintain Cdx2/Elf5 co-expression was evident (Fig. 6c), which correlated with a much more divergent cell size composition of colonies (with some cell remaining significantly smaller than TS cells, and others differentiating into giant cell-like cells). Even factors for which mRNA levels were close to TS cells exhibited an aberrant subcellular distribution. For example, the GPI-anchored Plet1 protein did not adopt the membrane-associated localization characteristic of TS cells but was distributed at lower protein levels in a predominantly cytoplasmic, speckled pattern as found in differentiating trophoblast (Fig. 6d).

**Figure 7:**
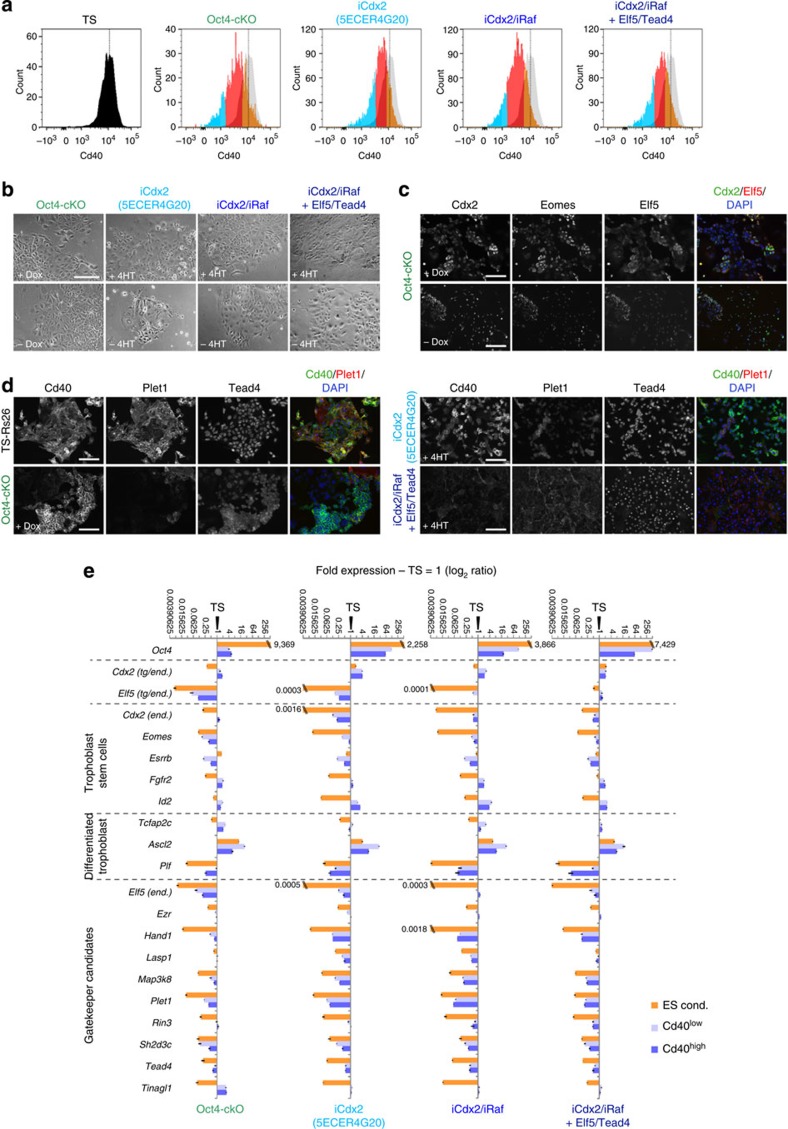
Enrichment for TS-like Cd40-high expressing cells does not select for stably converted iTS cells. (**a**) Fluorescence-activated cell-sorting strategy based on expression levels of the TS cell surface marker Cd40, as in Fig. 2. Cd40^low^ (cyan) and Cd40^high^ (orange) populations were purified after 6 days of reprogramming on MEFs. FACS profiles of TSL models are overlaid with that of TS cells (grey) and the median TS cell values indicated by the dotted line. Note that Cd40 in TSL models never reaches the Cd40^high^ profile characteristic of genuine TS cells, as shown in Fig. 2g. (**b**) Phase contrast images of Cd40^high^ cells replated for 7 days in the presence or absence of inducer (Dox or 4HT). Note the overall lack of epithelial integrity. Oct4-cKO cells appeared most TS-like in this system. Scale bar: 200 μm. (**c**) Cdx2, Eomes and Elf5 triple immunostaining on Oct4-cKO Cd40^high^-purified cells replated for ~4 weeks indicates loss of co-expression of these core TS cell transcription factors. Elf5 expression was patchy and remained well below that of TS cells. Scale bars: 100 μm (upper row), 200 μm (lower row). (**d**) Immunostaining for Cd40, Plet1 and Tead4 on Cd40^high^ selected cells as in **c**. Cd40 expression is lost, in particular in the iCdx2/iRaf+Tead4/Elf5 cells, and is patchy in the other TSL models after this culture period. Plet1 mostly fails to adopt membrane-bound localization at TS-equivalent levels in all models. Scale bars: 100 μm. (**e**) RT–qPCR survey of trophoblast markers and gatekeeper genes in reprogramming models grown in ES cell conditions as control, and in Cd40^high^ and Cd40^low^ sorted cell populations. Note the persistent imbalance of stable trophoblast gene expression and the failure to adequately downregulate Oct4 in many of these reprogramming models. Values are of three replicates and expressed as mean±s.e.m.

In a separate selection strategy, we sorted Cd40-high (and –low) cells after the initial 6-day reprogramming phase (Fig. 7a) and assessed these cells for morphological characteristics and gene expression profiles by immunostaining and reverse transcription–quantitative PCR (RT–qPCR; Fig. 7b–e). Overall, Cd40-high cells tended to lose most epithelial characteristics (Fig. 7b) and failed to stabilize Elf5 and Eomes expression (Fig. 7c), upregulate membrane-localized Plet1 and maintain Cd40 (Fig. 7d). This correlated with a global failure to acquire stable TS cell gene expression patterns (Fig. 7e).

**Figure 8:**
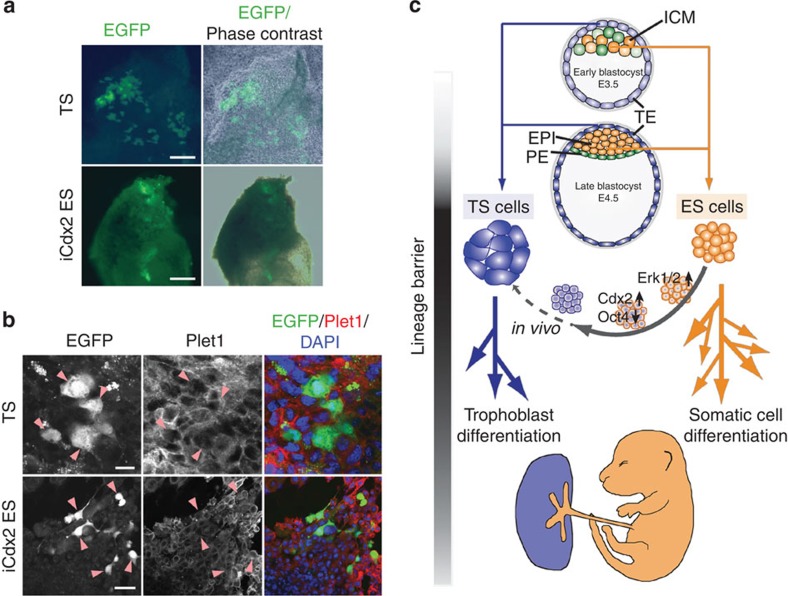
Chimerization potential of TSL cells. (**a**) Example overview images of a TS cell and iCdx2 ES cell chimera. Both cell lines carry constitutively expressed EGFP transgenes for identification. Please note, for better visualization of cellular contribution, the trophoblast compartment was dissected away from the embryo and flattened under a coverslip for the TS cell chimera. Scale bars: 100 μm (TS) and 250 μm (iCdx2). (**b**) Confocal images of outgrowths of chimeric trophoblast tissue stained for Plet1 (and Ezrin, shown in Supplementary Fig. 7). Arrowheads highlight chimerizing, EGFP-positive cells that are seemingly fully integrated into the tissue context and express Plet1 in a pattern indistinguishable from surrounding nonchimeric trophoblast cells. Scale bars: 20 μm (TS) and 50 μm (iCdx2). (**c**) Model of the profound divergence between the first two cell lineages in development and its epigenetic reinforcement that underpins the resistance to reprogramming in ES-to-TS cell conversion approaches. Some progression of reprogramming may occur in the niche environment *in vivo*, but on the whole ES-to-TS cell reprogramming remains incomplete *in vitro* and cells retain a reduced chimerization potential even *in vivo.* EPI, epiblast; ICM, inner cell mass; PE, primitive endoderm; TE, trophectoderm.

Finally, we investigated whether these partially reprogrammed TSL cells could complete the reprogramming process *in vivo*. For this purpose, we generated chimeras with TS-EGFP cells to establish a baseline contribution rate of bona fide TS cells, as well as with iCdx2 (5ECER4G20) and iCdx2/iRaf ES cells after induction of TSL differentiation on MEFs. In line with previous reports, iCdx2 and iCdx2/iRaf cells were indeed capable of colonizing the trophoblast compartment *in vivo* but did so at overall much lower frequencies (Table 1 and Supplementary Fig. 7). Of particular note was the reduced capacity to contribute in larger cell numbers, in line with the proliferative deficit of TSL cells that we had observed *in vitro* (Table 1; Fig. 8a). However, those cells that were chimerizing the trophoblast compartment had seemingly integrated fully into the tissue context and expressed epigenetically regulated markers such as Plet1 and Ezrin in a pattern indistinguishable from surrounding cells (Fig. 8b and Supplementary Fig. 7). Thus, individual TSL cells were indeed able to continue the reprogramming process *in vivo*, but on the whole TSL cells remained distinct from bona fide TS cells even in the *in vivo* context in terms of chimerization and clonal expansion potential.

## Discussion

Studying reprogramming in the context of ES and TS cells is unique as these two stem cell types reflect the earliest differentiation event after fertilization in which the future placental cells are set aside from all other somatic cells and the germ line. Thus, ES-to-TS cell reprogramming targets a cell lineage that is not ordinarily addressed in ‘conventional’ iPS cell generation strategies. Yet ES cells are the most developmentally plastic of all stem cell types, and hence should be the most amenable to reprogramming, including into the extraembryonic trophoblast lineage^45^.

Indeed, the generation of cells with TS-like features from ES cells has been reported. Remarkably, in the mouse this transition can seemingly be induced by overexpression of one single transcription factor, Cdx2 (ref. 18), or even by selection of specific ES cell states^46^,^47^. Perhaps most intriguing was the notion that TS cells can be derived from ES cells by constitutive activation of the Ras GTPase, thus placing signalling cascades at the pinnacle of cell fate-determining transcriptional networks^25^. Despite these seemingly straight-forward stem cell conversion strategies, ES and TS cell are separated by a fundamentally different epigenomic make-up characterized by unique histone and DNA methylation profiles^16^,^37^. Whether or not these defining patterns are fully reset in ES cells reprogrammed into iTS cells has not been investigated to date. Thus, we set out to assess how reprogramming between these two earliest stem cell types progresses to gain insights into the setting and resetting of lineage-specific epigenetic hallmarks.

As expected, all our ES-to-TSL reprogramming models acquired TS-like characteristics entirely in line with what has been reported before. Yet our in-depth analysis revealed that even in the most efficient reprogramming models, lineage transition remains incomplete and does not result in cells that are phenotypically, transcriptionally, epigenetically or functionally identical to bona fide TS cells.

Overall, our ES-to-TS cell reprogramming approaches reveal similarities, but also profound differences, to iPS cell reprogramming. In common with iPS cells is the retention of an epigenetic memory^48^,^49^,^50^, which led us to catalogue a number of key loci that represent defining hallmarks of lineage origin and stem cell type. We identify a small number of lineage ‘gatekeeper’ loci that are, like *Elf5*, unmethylated and expressed in TS cells but methylated and repressed in ES cells; these loci are particularly refractory to reprogramming, thus reinforcing the existing lineage barrier. Specifically targeting a select cohort of these factors does indeed improve reprogramming to some extent, as it does in iPS cells^51^, but still does not yield cells equivalent to bona fide TS cells even upon selection for TS-like features. The reasons for this could be (i) that expression of additional gatekeepers is needed to reach a TS-equivalent state; (ii) that precise levels and kinetics of expression or activation of the transcription factors and signalling pathways are instrumental to maintain the stem cell state of TS cells; and/or (iii) that other components such as microRNAs contribute to the retention of an epigenetic memory in these cells^52^.

It is interesting to note that this memory is retained also upon global hypomethylation by 2i treatment prior to the induction of reprogramming; thus, even ground-state pluripotent ES cells fail to undergo a complete ES-to-TS cell reprogramming process. While hypomethylation by 2i occurs on a genome-wide scale, some few loci, notably *Elf5* and *Ezr*, are partially spared. They retain DNA methylation at levels similar to those observed at imprinted genes (Supplementary Fig. 4a) and hence may share with imprinted genes some aspects of their epigenetic regulation^23^,^24^,^36^. It is further noteworthy that the reprogramming process did not improve with extended culture periods. In contrast to observations in iPS cells, however, the methylation changes that we observed during the trans-differentiation process remained canalized within the boundaries set by ES and TS cells, with no aberrant methylation hotspots detected that are not present in either ES or TS cells^49^.

Our methylation profiling revealed a number of loci (subcluster ‘A’ in Fig. 3c) that are readily susceptible to methylation changes in response to activation of Erk1/2 signalling. These loci may be particularly relevant in the context of assisted reproductive technologies (ART) where culture conditions may well overstimulate signalling pathways such as the Erk1/2 cascade, which may consequently lead to heritable epigenetic changes. Since our reprogramming models reflect the earliest cell fate decision that occurs in the preimplantation period, these results are directly relevant for this critical developmental time window that is affected by ART procedures. Overall, however, our data highlight that DNA methylation dynamics are highly dependent on the genomic context, an observation likely tied to the regulatory elements that control these DNA regions. It has been noted before that steroid hormone-responsive promoters undergo cyclical changes of DNA methylation, and also Fgf2-induced effects on local chromatin modifications have been reported^53^,^54^,^55^. These examples emphasize the need for integrating DNA methylation dynamics in a locus- and signalling network-dependent manner.

Perhaps the greatest impact of our work is on the notion of reprogramming between the first two cell lineages. Our in-depth assessment revealed that the induced cell fate switch remains incomplete even in the seemingly best ES-to-TS cell reprogramming models. Remarkably, these differences—perpetuated through a retained epigenetic memory of the ES cell origin and resulting in a labile and inadequate trophoblast-like transcriptional programme—remain functionally relevant even *in vivo*. Thus, upon reintroduction into chimeras, reprogrammed TS-like cells exhibited a lower chimerization potential as well as a markedly reduced clonal contribution rate compared with bona fide TS cells. At the same time, however, individual TSL cells chimerizing the trophoblast compartment seemed to be capable of continuing the reprogramming process as they re-expressed epigenetically regulated gatekeeper genes such as *Plet1* and *Ezr* in a pattern indistinguishable from surrounding, non-chimeric trophoblast cells. Such an expansion of cellular reprogramming capacity within niche environments *in vivo* has gained significant support recently. Indeed, reprogramming *in vivo* seems to advance much more extensively than *in vitro*, and includes the resetting of DNA methylation patterns^56^, and even the acquisition of totipotent features of cells in which the four ‘Yamanaka’ factors are activated^57^. While reprogramming of individual TSL cells may be enhanced *in vivo* within the trophoblast compartment in a similar manner, our results demonstrate that the ES-to-TS cell lineage conversion is not easily brought to completion (Fig. 8c). Upon closer examination, our results are supported by multiple other findings: (i) the transcriptome of Oct4-cKO and iCdx2 ES cells is similar, but not identical, to that of bona fide TS cells^34^,^58^; (ii) the genome-wide occupancy maps of the transcription factor Sox2 show only a ~50% overlap between embryo-derived TS cells and Oct4-cKO-derived TSL cells; and (iii) the various TSL models generally rely on the persistent presence of environmental factors provided by a MEF feeder layer^17^,^18^,^19^,^25^,^34^. As such, the major stumbling block to complete lineage conversion appears to be the maintenance of epithelial integrity combined with the retention of self-renewal capacity of TSL cells. Both aspects fail because of fluctuating and unstable expression levels of TS cell factors, indicating that the resetting of the epigenome to re-establish stable transcriptional programmes has not been fully accomplished. Thus, while TSL cells derived from ES cells may be beneficial for specific research questions as model systems, they should be regarded with caution as their resemblance to TS cells is tenuous and labile. The same caveats hold true for trophoblast-like cells derived from human ES cells^59^. Our various reprogramming strategies demonstrate that, in fact, the first cell lineage divergence in development is so profound that ES cells are highly resistant to reprogramming into genuine TS cells, *in vitro* and even *in vivo*. Presumably, these tight lineage-reinforcing barriers serve to protect the embryo from ectopic differentiation of trophoblast derivatives that would have fatal consequences on its development because of the invasive, pro-angiogenic and haemorrhagic properties of trophoblast^60^.

## Methods

### Stem cell culture

ES and TS cells were cultured in routine conditions as described previously^16^. As control lines, ES E14tg2a and J1 cells (obtained from the Mutant Mouse Regional Resource Center, UC Davis, USA, and the American Type Culture Collection-Laboratory of the Government Chemist (ATCC-LGC) Standards, Middlesex, UK) and blastocyst-derived TS-EGFP and Rs26 cells (both a kind gift of the Rossant lab, Toronto, Canada; mixed ICR × 129 genetic background) were used. Zhbtc4 Oct4-cKO cells and 5ECER4G20 EGFP:Cdx2:ER ES cells (E14tg2a background) were a kind gift from H. Niwa (Kobe, Japan)^18^,^21^; we also tested a Dox-inducible iCdx2 cell line generated in KH2 ES cells (kindly obtained from the Schorle lab, Bonn, Germany) that yielded very similar results as the iCdx2 cells generated by us^19^. ES-E14 cells were used to generate stably transfected TSL lines with the following constructs: pCAG-IRES-EGFP (‘control ES’), pCAG-ER:Hras^G12V^-IRES-EGFP (‘iRas’), pCAG-ΔRaf-1:ER-IRES-EGFP (‘iRaf’) and pCAG-Cdx2:ER-Puro^18^ (‘iCdx2’). iRas consisted of the hormone-binding domain of the oestrogen receptor (ER) fused to the GTPase-deficient HRas^G12V^ oncogene (ER-HRas^G12V^), while iRaf consisted of the kinase domain of Raf-1 fused to ER (ΔRaf-1:ER*). Further manipulations were performed with piggyBac vectors^61^ as described. The complete open reading frames were cloned for all genes except *Tead4*, for which the DNA-binding domain fused to the transcriptional activation domain of herpes simplex virus VP16 was used, as described previously^17^. All factors cloned were sequence-verified. Induction of trophoblast differentiation was performed by plating 10^4^ cells into T25-cm^2^ flasks in TS cell media on a layer of irradiated MEFs and in the presence of 1 μg ml^−1^ 4HT, unless stated otherwise.

### Immunofluorescence staining

For immunofluorescence staining, cells were grown on coverslips, fixed with 4% paraformaldehyde, permeabilized and blocked with 0.25% bovine serum albumin and 0.1% Tween-20 in PBS (PBT/BSA). Primary antibodies and dilutions used were mouse anti-Cdx2 1:400 (Biogenex MU392A-UC), goat anti-Elf5 1:200 (Santa Cruz sc-9645), mouse anti-Oct4 1:400 (Santa Cruz sc-5279), goat anti-Cd40 1:100 (R&D Systems AF440), rabbit anti-Eomes 1:400 (Abcam ab23345), mouse anti-Tead4 1:400 (Abcam ab58310), rat anti-Plet1 1:100 (MUbio MUB1512P) and rabbit anti-Ezrin 1:100 (Cell Signalling no. 3145), detected with appropriate secondary AlexaFluor 488, 568 or 647 antibodies. Cells were counterstained with 4',6-diamidino-2-phenylindole (DAPI) and observed using an Olympus BX41 or BX61 epifluorescence microscope or a Zeiss LSM 780 confocal microscope.

### Flow cytometry

ES cells undergoing trans-differentiation were collected and analysed at regular intervals for cell surface antigens specific for ES cells (Cd31/Pecam1; BD 551262), TS cells (Cd40; R&D AF440, conjugated to PE-Cy7) and XEN cells (Cd140a/Pdgfra; eBioscience 12-1401-81)^35^, as well as MEFs (Miltenyi Biotech 130-096-095). Dead cells were discriminated by 7-amino-actinomycin D (eBioscience 00-6993-50). Cells were harvested in 0.05% trypsin/EDTA (Invitrogen 25300-054) plus 2% chicken serum, washed, gently resuspended, filtered through a 40-μM cell strainer and resuspended in 2% fetal bovine serum in PBS. Sequential stainings were performed at 4 °C, by incubation with primary and secondary antibodies for 30 min each. Cells were resuspended in a final volume of 500 μl of PBS/2% FCS (supplemented with 1% 7-amino-actinomycin D) and analysed with a Becton Dickinson LSRII flow cytometer. At least 10,000 live cells were measured for each sample/condition. Raw data sets were analysed using the FlowJo software. Fluorescence-activated cell sorting (FACS) for EGFP-positive cells was performed using a Becton Dickinson FACSAria cell sorter.

### Alkaline phosphatase staining

Alkaline phosphatase assay was performed using a commercial kit according to the manufacturer’s instructions (Sigma 86R-1KT).

### Western blotting

Whole-cell extracts were prepared with TG buffer (20 mM Tris-HCl, pH7.5, 137 mM NaCl, 1 mM EGTA, 1% Triton X-100, 10% glycerol and 1.5 mM MgCl_2_) supplemented with protease inhibitor cocktail (Sigma P2714) and phosphatase inhibitors (50 mM NaF and 1 mM Na_3_VO_4_). Protein lysates (20 μg) were resolved by sodium dodecyl sulfate polyacrylamide gel electrophoresis (SDS–PAGE) and transferred using a Bio-Rad Mini Trans Blot system (170-3930) on polyvinylidene difluoride membrane (Immobilon-P, Millipore). Membranes were blocked with 5% milk powder or BSA and incubated with specific primary antibodies overnight at 4 °C, followed by horseradish peroxidise-conjugated secondary antibodies. Detection was carried out with enhanced chemiluminescence reaction (GE Healthcare RPN2209) on standard X-ray films. Antibodies used are given in Supplementary Table 1, and all primary scans of blots are shown in Supplementary Fig. 8).

### RT–qPCRs

Total RNA was extracted using TRI reagent (Sigma T9424) according to the manufacturer’s instructions, and any potential DNA contamination was removed either by treatment with DNaseI (NEB M0303) in the presence of RNase inhibitors (Fermentas EO0381) or with the TURBO DNA-free kit (Life Technologies AM1907) according to the manufacturer’s instructions. One to two micrograms of total RNA were used for cDNA synthesis with RevertAid H-minus M-MuLV Revert Transcriptase (Fermentas EP0451) and random hexamers (Promega C118A).

Quantitative PCR was performed using SYBR Green Jump Start Taq Ready Mix (Sigma S4438) on a Bio-Rad CFX96 thermocycler. All primer pairs were intron-spanning and are provided in Supplementary Table 2.

### DNA methylation analysis

ES cells undergoing trans-differentiation were FACS-purified to exclude MEFs, lysed in 100 mM Tris-HCl, pH 8.5, 5 mM EDTA, pH 8.0, 0.2% SDS, 200 mM NaCl, 200 μg ml^−1^ Proteinase K at 60 °C overnight and the DNA purified by phenol–chloroform extraction. One to two micrograms of DNA were used for bisulphite conversion using the EpiTect kit (Qiagen 59104) according to the manufacturer’s instructions. Genomic regions of interest were amplified in simple or nested PCRs, products cloned into pGEM-T Easy Vector system (Promega) and 8–10 colonies per sample picked, and plasmids extracted (QIAprep Spin Miniprep Kit, Qiagen) and sequenced. Nonclonality of sequenced alleles was confirmed. Alternatively, amplified bisulphite PCR products were processed for analysis by Sequenom MassArray technology according to standard protocols^62^ and manufacturer’s instructions.

### Global DNA methylation and expression profiling

MeDIP-seq was performed as described previously^16^,^63^. Briefly,~3 μg genomic DNA were sonicated using a Diagenode Bioruptor UCD-200 to 200–700 nucleotide (nt) fragments, DNA-end repaired, dA-tailed and ligated to paired-end adaptors using the NEB Next DNA Library Prep Master Mix set (NEB E6040); surplus adaptors were removed using Agencourt AMPure XP SPRI beads (Beckman Coulter, A63881). Immunoprecipitations (IPs) were performed in triplicate for each cell type with 500 ng DNA/sample with an anti-5mC antibody (Eurogentec BI-MECY-0100). IPs were carried out with sheep anti-mouse IgG Dynabeads M-280 (Invitrogen 112-01D). Triplicate IPs were pooled and column-purified (MinElute, Qiagen). MeDIP libraries (and unbound DNA fractions, as control) were PCR-amplified and bar-coded (iCdx2, iCdx2/iRaf and 2i-pre-treated TSL/ES cells). 5mC IP efficiency was confirmed by analysing ES and TS cell meDIP pre- and post-amplification libraries by quantitative PCR for the *Nanog* and *Elf5* loci. Massive parallel sequencing was performed either on an Illumina GAIIX Genome Analyser (individual libraries) or an Illumina HiSeq1000 sequencer for bar-coded libraries. In all, 15–35 × 10^6^ reads were obtained from each meDIP library. Genomic mapping of paired-end reads was performed with Bowtie (V0.12.8)^64^ using the following parameters: -m 1 –strata –best –X 700. Reads were mapped to the mouse genome build NCBIM37. Final data analysis was performed using the SeqMonk software (www.bioinformatics.babraham.ac.uk).

Probe read counts were quantified and subjected to multistep normalization—involving corrections for total read count and 75% percentile distribution, followed by forced matching of distributions. Functional annotation of DNA sequences was performed using DAVID^65^.

RNA-seq was performed on cells after an initial 5-day reprogramming phase on MEFs, and after replating for 3 days on gelatinized tissue culture dishes. Cells were FACS-purified to separate them from MEFs, and total RNA prepared using TRI reagent (Sigma T9424) followed by DNase treatment using the TURBO DNA-free kit (Life Technologies AM1907), according to the manufacturers’ instructions. mRNA was isolated from total DNA-free RNA (150–240 ng) using the Dynabeads mRNA purification kit (Life Technologies 61006) and prepared into an indexed, strand-specific library using the ScriptSeq v2 RNA-Seq Library Preparation Kit (Epicentre SSV21106) according to the manufacturers’ instructions. Libraries were quantified/assessed using both the KAPA Library Quantification Kit (KAPA Biosystems KK4824) and Bioanalyzer 2100 system (Agilent). Indexed libraries were pooled and sequenced with a 100-bp single-end protocol. Raw fastq data were mapped to the *Mus musculus* NCBIM37 genome assembly using TopHat v2.0.12, guided by gene models from Ensembl v61. Data were quantitated at a protein-coding mRNA level using the RNA-seq quantitation pipeline in the SeqMonk software (www.bioinformatics.babraham.ac.uk) and normalized according to total read count (reads per million), 75% distribution and followed by forced matching of distributions. Differentially expressed gene lists were compiled through intensity difference filter (*P*<0.05). For identification of most meaningful expression changes as shown in Fig. 5e, data were compared with expression profiles of TS cells following MEK inhibition, a key signalling pathway essential for the maintenance of the stem cell state of TS cells.

### Chimera production

For the assessment of *in vivo* chimerization potential, constitutively EGFP-expressing TS cells, iCdx2 (5ECER4G20) ES and iCdx2/iRaf ES cells were used. ES cells were subjected to a 5-day reprogramming regime on MEFs, were FACS-purified and microinjected into E3.5 C57BL/6Babr or (C57BL/6Babr × CBA) × (C57BL/6Babr × CBA) F2 blastocysts. Embryos were transferred on the same, or the following day, into CD1 foster mothers; dissections and analyses were carried out at E7.5. All animal experiments were conducted in full compliance with UK Home Office regulations and with approval of the local animal welfare committee at The Babraham Institute, and with the relevant project and personal licences in place. For analysis of functional integration and enhanced reprogramming of chimeric cells, the trophoblast compartment was explanted into four-well dishes on coverslips and cultured for 2–3 days in standard TS cell medium. Immunofluorescence was carried out as above; samples were imaged at a Zeiss LSM 780 confocal microscope.

## Author contributions

F.C., A.M., W.D., D.D., C.E.S. and M.H. conducted experiments, F.C., A.M., F.K. and S.A. performed the bioinformatic analyses, and S.J.C. and M.H. designed the study, interpreted data and wrote the manuscript.

## Additional information

**How to cite this article:** Cambuli, F. *et al*. Epigenetic memory of the first cell fate decision prevents complete ES cell reprogramming into trophoblast. *Nat. Commun.* 5:5538 doi: 10.1038/ncomms6538 (2014).

**Accession codes**: High-throughput meDIP-seq and RNA-seq data sets have been deposited with the following accession codes: SuperSeries GSE62150; individual accession numbers are: MeDIP data (GSE62138) and RNA-Seq data (GSE62149).

## Supplementary Material

Supplementary Information Supplementary Figures 1-8, Supplementary Tables 1 and 2

## Figures and Tables

**Table 1:** Summary of chimera results.

	**Embryos recovered**	**Average % chimeric**	**% Contribution >10 cells**
TS-EGFP	32	48	16
iCdx2 (5ECER4G20) ES	27	28	4
iCdx2/iRaf ES	39	23	3

EGFP, enhanced green fluorescent protein; ES, embryonic stem cell; TS, trophoblast stem cell.
