# Supplementary material for: Epigenetic memory of the first cell fate decision prevents complete ES cell reprogramming into trophoblast
Source: Nat Commun. 2014 Nov 26;5:5538. doi: 10.1038/ncomms6538 (PMC4263130; doi:10.1038/ncomms6538)
Supplement: Supplementary Information — Supplementary Figures 1-8, Supplementary Tables 1 and 2 [file ncomms6538-s1.pdf]

Supplementary Figure 1

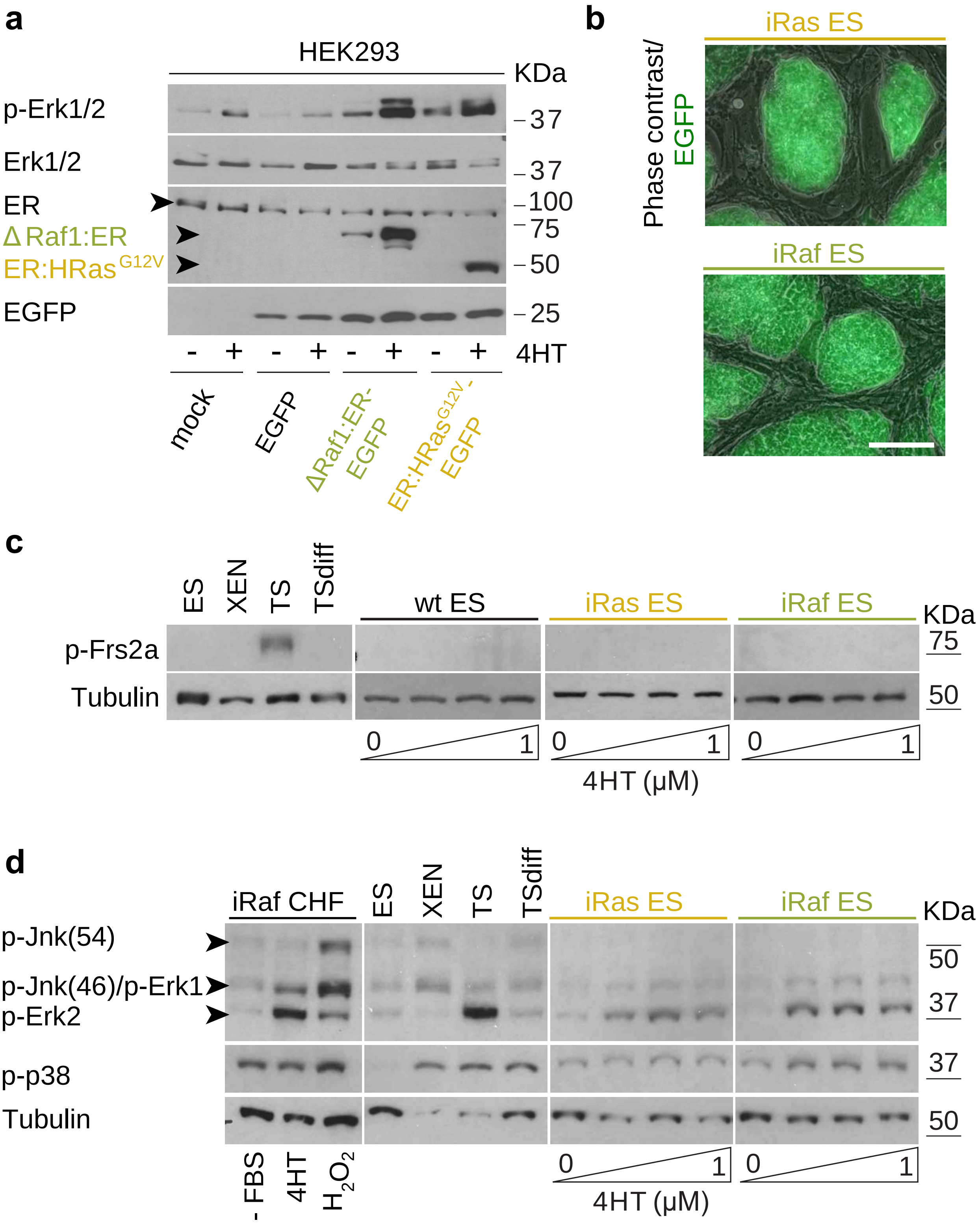

Supplementary Figure 1: Establishment of iRas and iRaf mouse ES cell lines.

(a) Proof of functionality of inducible Ras and Raf estrogen receptor fusion proteins with estrogen receptor (ER) in HEK293 cells. Note that these constructs also drive EGFP expression from an internal ribosomal entry site (IRES) downstream of the Ras/Raf:ER fusion protein. Upon exposure to 4-hydroxytamoxifen (4HT), these fusion proteins become de-repressed and stabilized and confer high levels of phosphorylation, and hence activation, of Erk1/2 (p-Erk1/2; also known as Mapk3 and Mapk1). **(b)** Homogenously EGFP-positive stable ES cell colonies were picked, expanded, and resulting lines with p-Erk1/2 activation levels comparable to those in TS cells were selected for both constructs. Scale bar: 100 µm. **(c)** The specificity of the 4HT-induced intracellular signalling was confirmed by monitoring phosphorylation of Frs2α, an adaptor protein involved in propagating fibroblast growth factor receptor (Fgfr) signalling upstream of Ras, and the Jnk and p38 kinases (in (d)), that are analogous to Erk1/2 but activated predominantly by cytokines and stress signalling. Phosphorylation of the Fgfr2 adaptor protein Frs2a is observed in TS cells, but not in any of the other stem cell lines or in the iRas or iRaf ES cells upon 4HT exposure. **(d)** Assessment of Jnk and p38 activation in iRas and iRaf models. Note that phosphorylated p38 (p-p38) is absent from ES cells; a modest level of activation is observed in iRaf ES cells upon prolonged 4HT exposure.

Supplementary Figure 2

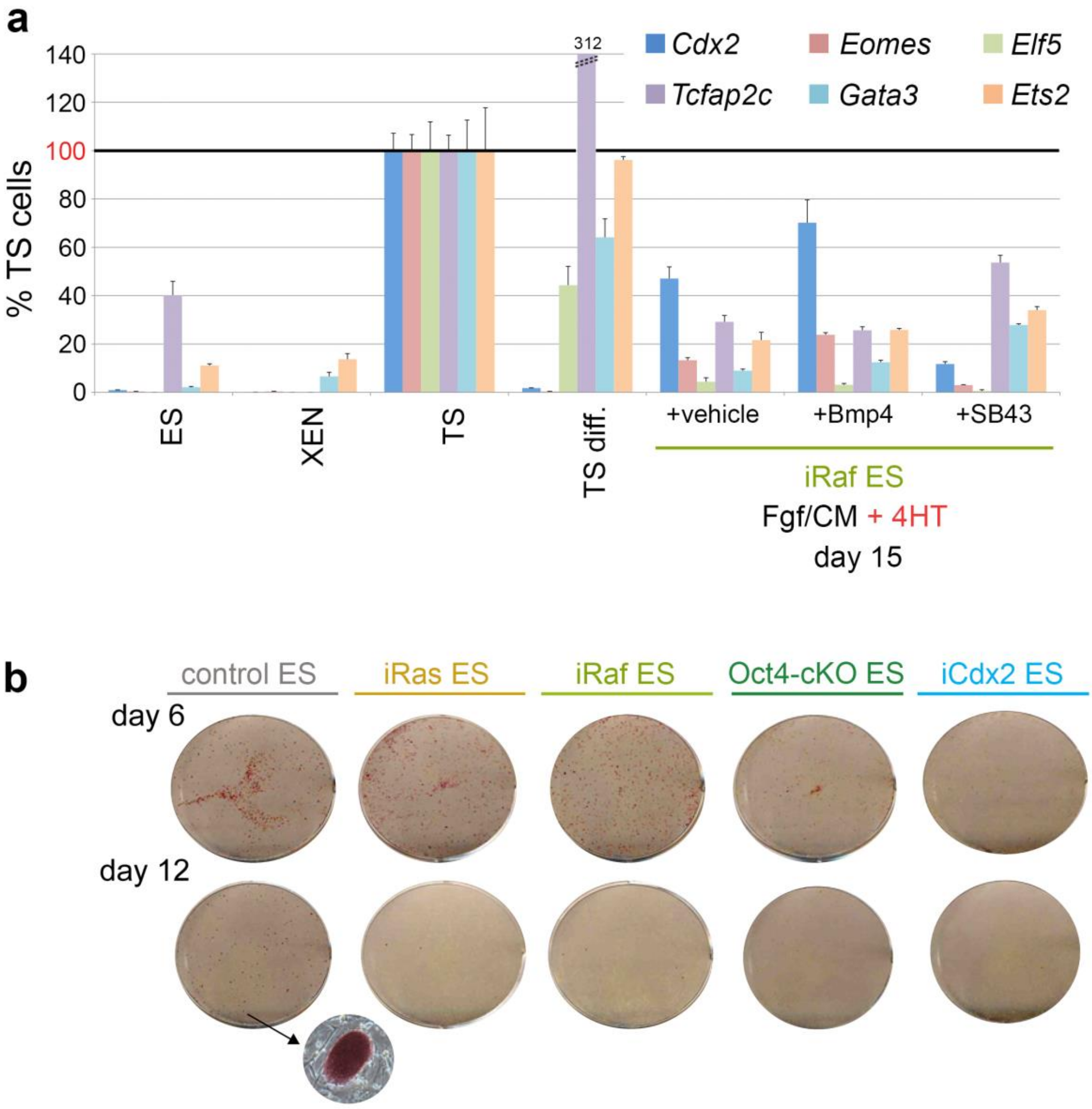

Supplementary Figure 2: Additional assessment of ES-to-TS-like reprogramming models. **(a)** iRaf ES cells 15 days after the beginning of trans-differentiation in the presence or absence of Bmp4 or the Activin receptor inhibitor SB431542. Bmp4 treatment induced a marked up-regulation of *Cdx2*, but did not result in increased expression of any other member of the TS cell core transcriptional network. Inhibition of Smad signalling by the specific Alk4/5/7 receptor inhibitor SB431542 resulted in down-regulation of TS-cell transcription factors which predominantly sustain self-renewal (*Cdx2, Eomes, Elf5*) and up-regulation of those also involved in the initial phase of trophoblast differentiation (*Tcfap2c, Gata3, Ets2*). All RT-qPCR expression data are shown as mean ± s.e.m. of three biological replicates. **(b)** Monitoring the kinetics of exit from self-renewal by alkaline phosphatase (AP) staining revealed a rapid loss of the stem cell state during the trans-differentiation process of Oct4-cKO and iCdx2 cells that was complete before day 6, while iRas and iRaf TSL cells exhibited a more protracted loss of AP activity only by day 12.

Supplementary Figure 3

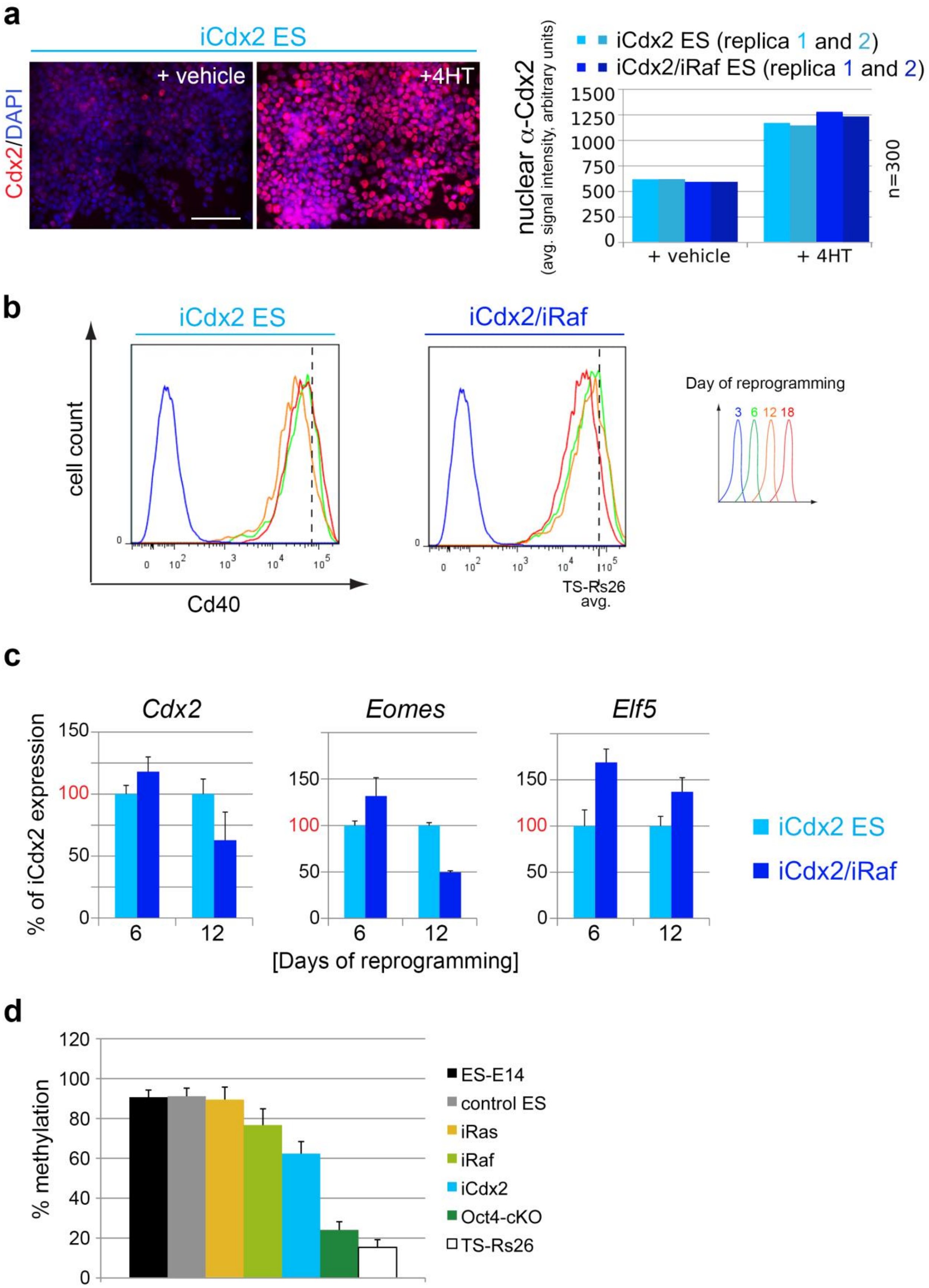

Supplementary Figure 3: Establishment and comparative analysis of iCdx2 and combined iCdx2/iRaf ES cells.

(a) iCdx2 and iCdx2/iRaf ES cell lines were established by analysis of nuclear Cdx2 immunofluorescence staining intensity upon 4HT exposure using the BD Pathway 855 semi-automated clone screening system. Clones with similar staining intensities, and lack of leaky expression in the absence of 4HT, were chosen. Scale bar: 100 µm. **(b)** Comparative analysis of iCdx2 and iCdx2/iRaf ES cells 12 days after the onset of trans-differentiation by Cd40 flow cytometry. No prominent difference in shift towards $Cd40^{high}$ expression levels was observed between both models. **(c)** Expression analysis of TS cell genes 6 and 12 days after TSL induction. Although iCdx2/iRaf ES cells exhibited slightly higher *Elf5* expression levels compared to iCdx2 cells, *Eomes* expression levels still remained unstable. Data are of three biological replicates and presented as mean ± s.e.m. **(d)** DNA methylation analysis at the *Elf5* promoter by Sequenom MassArray in the 4 reprogramming models. Note that methylation reprogramming at *Elf5* is more pronounced in Oct4-cKO than in iCdx2 cells. Data are of three biological replicates and presented as mean ± s.e.m.

Supplementary Figure 4

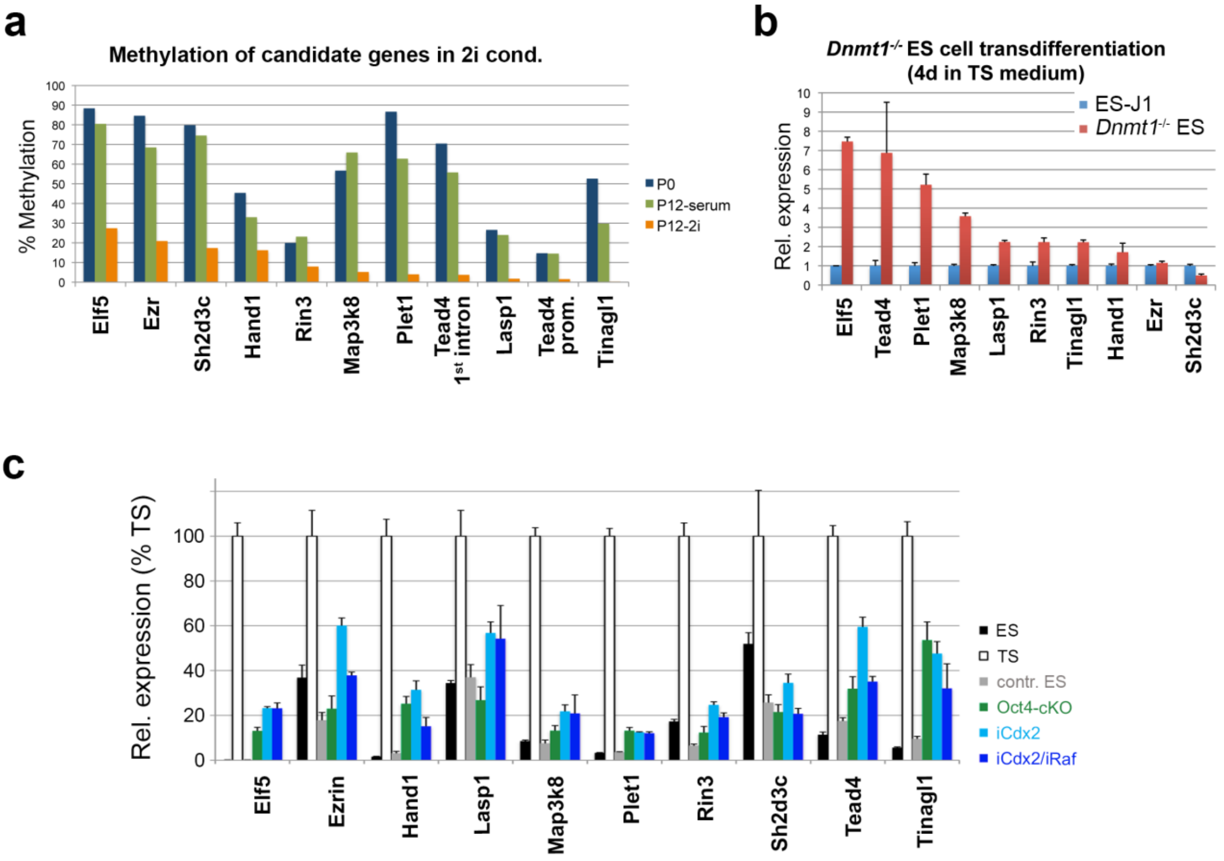

Supplementary Figure 4: Lineage gatekeeper loci under tight methylation control.
(a) Methylation levels of the identified gatekeeper genes (at the identified differentially methylated regions) in ES cells cultured either in standard serum conditions or in the presence of Mek1/2 and Gsk3 inhibitors ("2i") that causes global demethylation. Data are from Ficz et al. (Ref. 24). Retention of methylation marks to levels similar to those observed at imprinted genes is evident in particular at the *Elf5* locus, as well as at *Ezr*, *Sh2d3c* and *Hand1*. **(b)** Expression of the 10 identified differentially methylated genes between ES and TS cells in *Dnmt1*-deficient ES cells after 4 days of culture in TS cell conditions, during which a trophoblast expression programme is activated due to the globally hypomethylated background (Ref. 15). *Elf5* is the most strongly induced trophoblast marker, followed by *Tead4, Plet1* and *Map3k8*, indicating that perhaps these genes are particularly indicative of the onset of trophoblast differentiation (n=3; mean ± s.e.m). **(c)** Expression levels, determined by RT-qPCR, of the 10 gatekeeper genes in TSL models after 12 days of reprogramming, expressed relative to TS cell levels as mean ± s.e.m. of three biological replicates. ES-DMR associated "gatekeeper" genes fail to become activated in TSL models to TS-equivalent levels.

Supplementary Figure 5

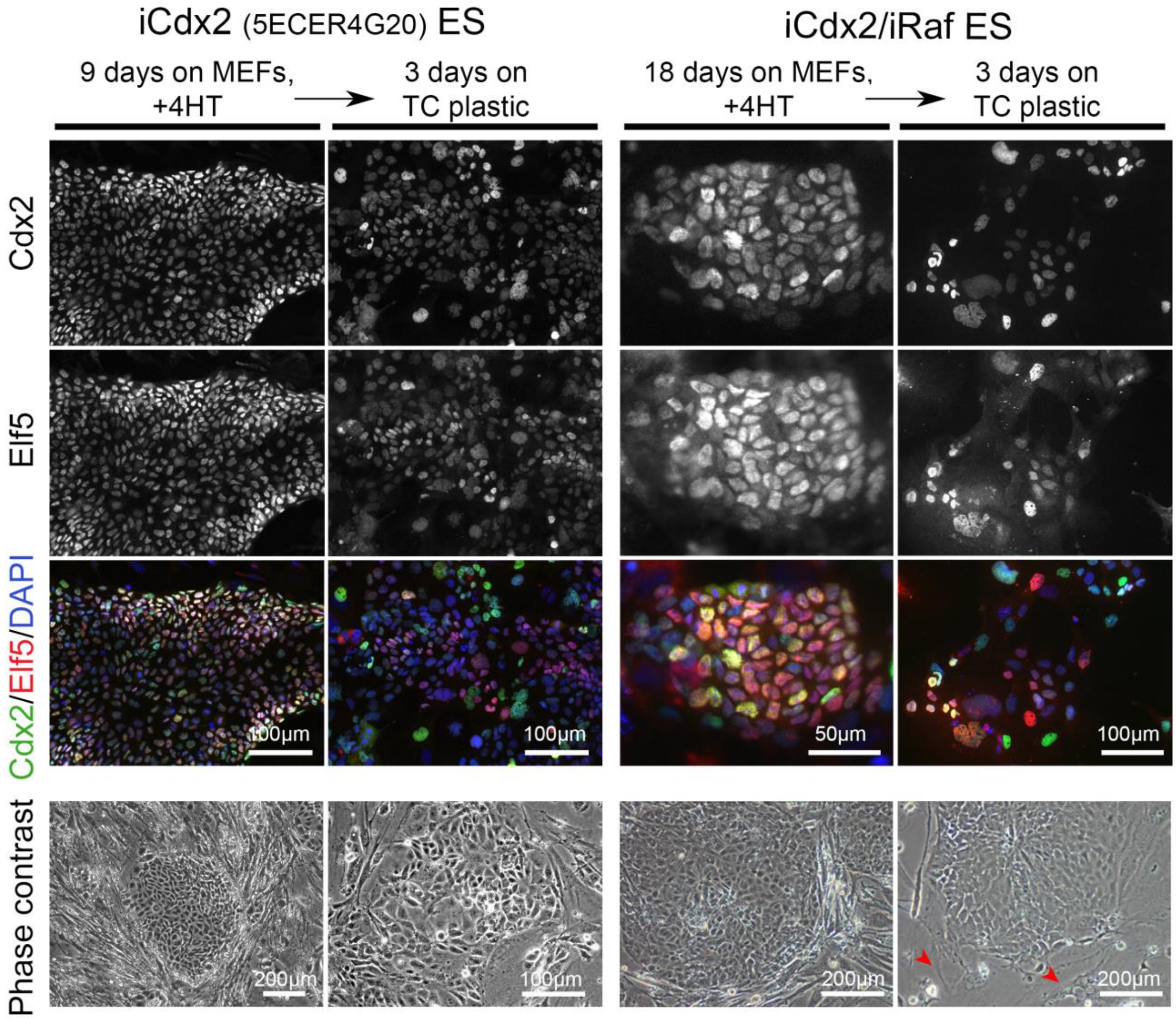

Supplementary Figure 5: Loss of phenotypic stability of reprogrammed TSL cells.

iCdx2 (5ECER4G20) cells containing a pCAG-EGFP:Cdx2:ER-IRES-Puro construct, an additional EGFP transgene and a targeted Oct4 allele, and iCdx2/iRaf cells were assessed for morphological and gene expression stability after 9 and 18 days of trans-differentiation on MEFs, and then again after an additional 3 days on regular tissue culture plastic. For clarity, single-channel images are shown in black and white. On MEFs, iCdx2 cells formed epithelial colonies very much akin to *bona fide* TS cells as previously reported, and they exhibited widespread expression of the trophoblast transcription factors Cdx2 and Elf5. Despite this TS-like appearance, however, the characteristically tight colony shape was rapidly lost when cells were plated in the absence of MEFs, and instead cells started to spread out and started to differentiate. Concomitantly, the expression of Cdx2 and Elf5 became de-regulated; Cdx2 was retained in giant cell-like cells at high levels, while Elf5 was present in central, more stem-like cells but was uncharacteristically rapidly lost in differentiating cells at the margins. Scale bars

are as indicated: 100 µm in immunofluorescence stainings columns 1,2 and 4, and 50 µm in column 3; 200 µm in phase contrast images columns 1,3, and 4, and 100 µm in column 2.

Supplementary Figure 6

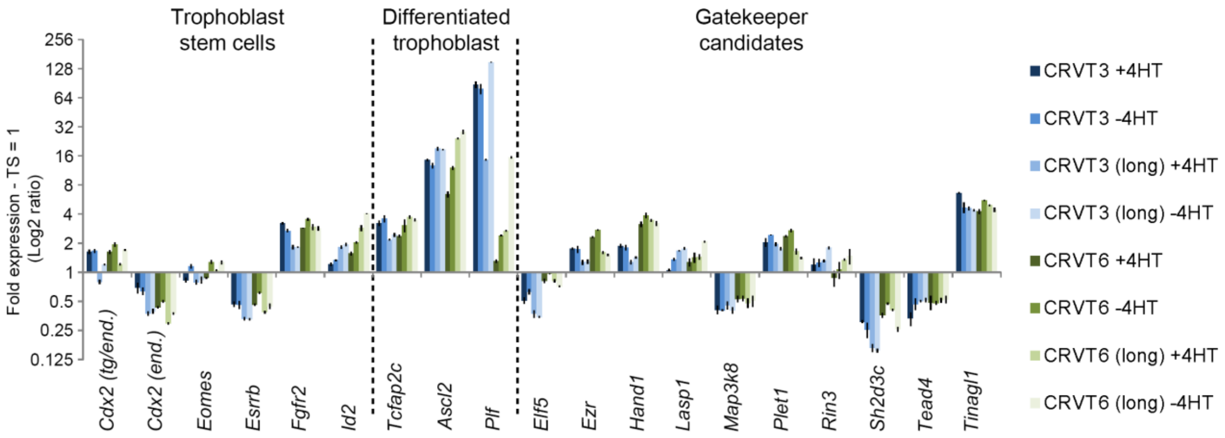

Supplementary Figure 6: The partially reprogrammed TSL phenotype is stable in long-term culture.

RT-qPCR analysis of iCdx2/iRaf clones CRVT3 and CRVT6, established by selection for most TS-like morphology and gene expression pattern, after continued propagation in the presence or absence of 4HT. Reprogramming was initiated on MEFs for 5 days, cells were then plated onto tissue culture plastic and passaged/grown in the presence of 4HT, during which time colonies were picked and expanded (all in presence of 4HT). After 5 weeks, cells were split and grown with or without 4HT for 2 days before collecting RNA (as in Figure 6b). CRVT (long) cells were grown for an additional 2-3 weeks in the presence or absence of 4HT before RNA collection. Long-term culture of these cells does not help improve the acquisition of a full TS-like transcriptional profile. The expression profiles of the marker and gatekeeper genes tested remains largely unchanged, indicating that the partial reprogramming phenotype is stable and does not further progress in culture under continued selection (n=3; data are presented as mean ± s.e.m).

Supplementary Figure 7

a

	Transfer	No. transferred	No. retrieved	No. chimeric	% chimeric	average % chimeras	No. with >10 cells contribution	% of chimeras w/ contr. >10 cells	% of all retrieved with contr. > 10 cells
TS-EGFP	1	7	6	4	67	48	2	50	16
	2	15	10	4	40		0	0	
	3	18	16	6	38		3	50	
iCdx2 (5ECER4G20)	1	10	9	3	33	28	1	33	4
	2	16	13	4	31		0	0	
	3	15	5	1	20		0	0	
	4	17	0						
iCdx2/iRaf	1	15	0			23			3
	2	14	11	3	27		0	0	
	3	16	14	3	21		1	33	
	4	16	14	3	21		0	0	

b

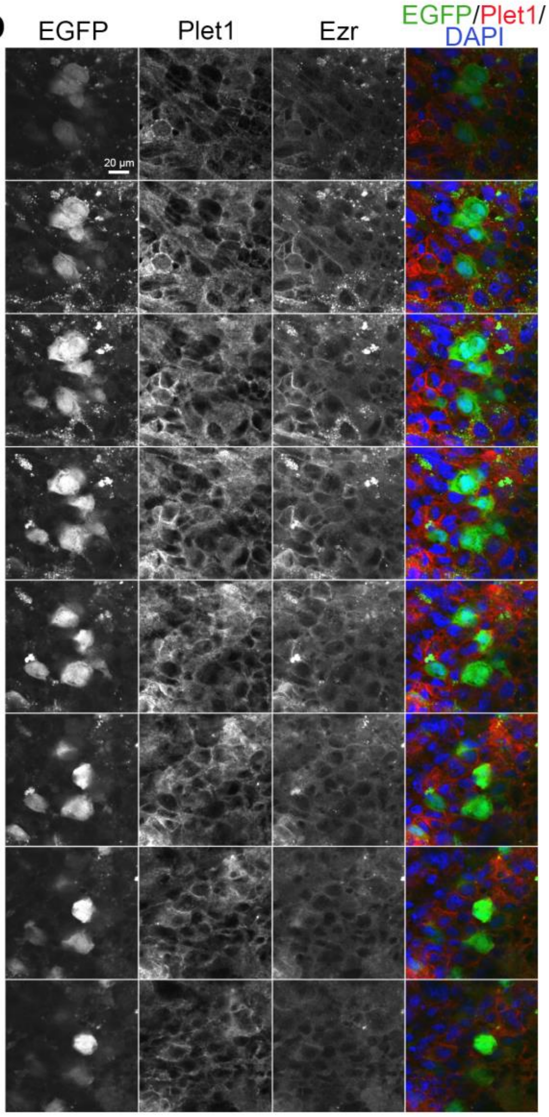

c

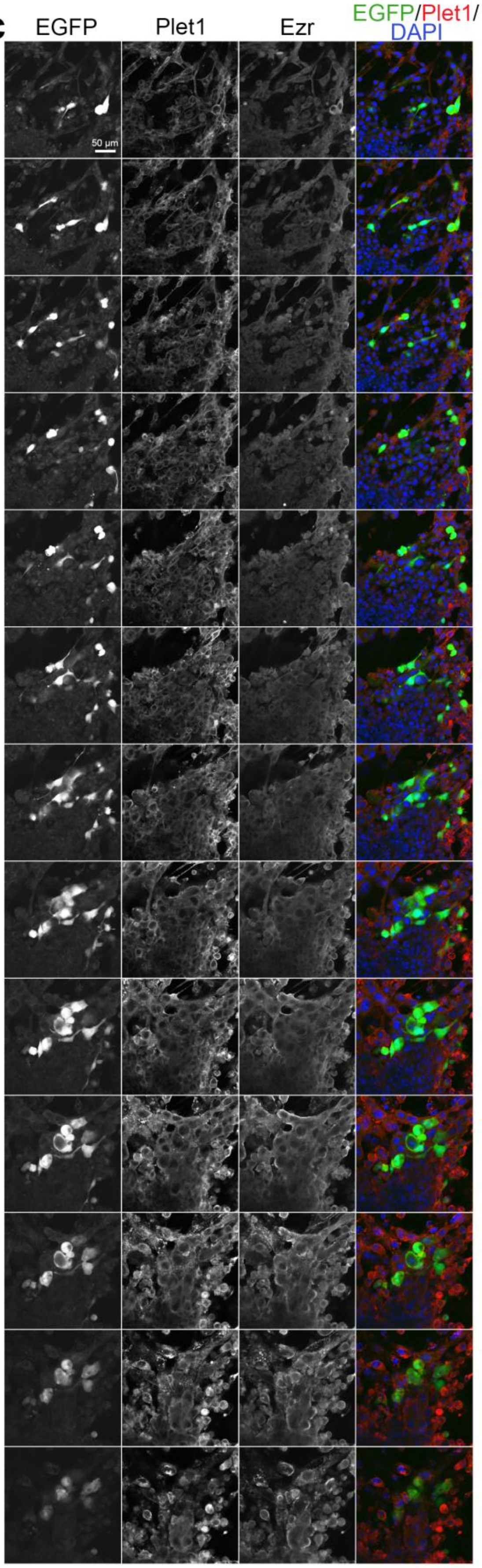

Supplementary Figure 7: Analysis of *in vivo* chimerization potential of TSL cells in comparison to *bona fide* TS cells.

(a) Details of blastocyst injection experiments to produce chimeras. TS-EGFP cells were used as controls to obtain chimerization potential values of genuine TS cells against which the experimental iCdx2 and iCdx2/iRaf TSL cells could be compared. iCdx2 and iCdx2/iRaf ES cells were induced for reprogramming for 4-5 days on MEFs in the presence of 4HT, and then used for blastocyst injection. **(b)** and **(c)** Full confocal stacks of trophoblast outgrowths of a representative TS cell chimera (b) and iCdx2 cell chimera (c), as shown in Figure 8. Outgrowths were cultured for 3 days in TS cell conditions, and then stained for Plet1 and Ezrin (Ezr). Scale bars areas indicated: 20 µm in (b); 50 µm in (c).

Supplementary Figure 8

(a) Primary scans of Western blots as in Figure 1a

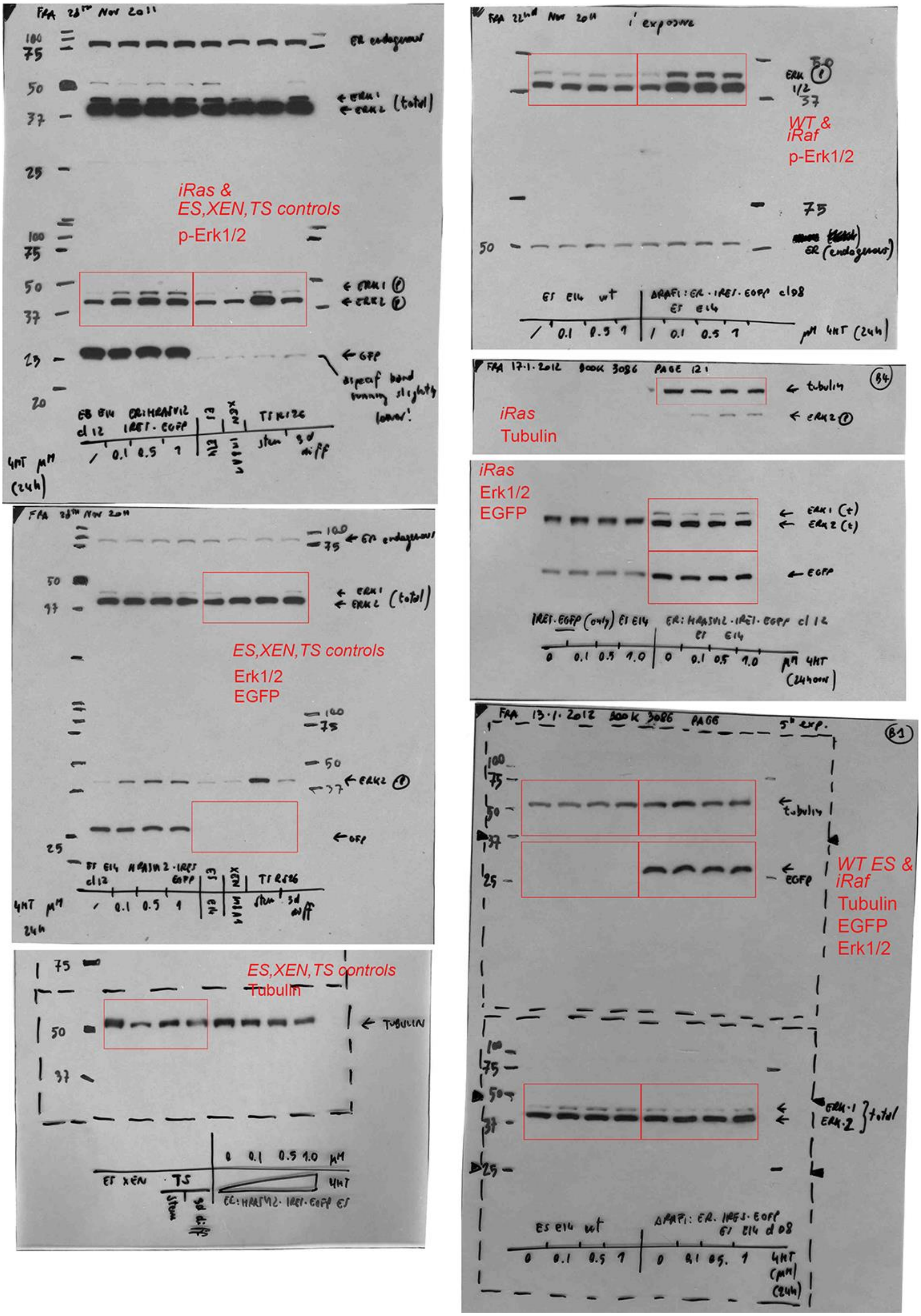

(b) Primary scans of Western blots as in Figure 1c

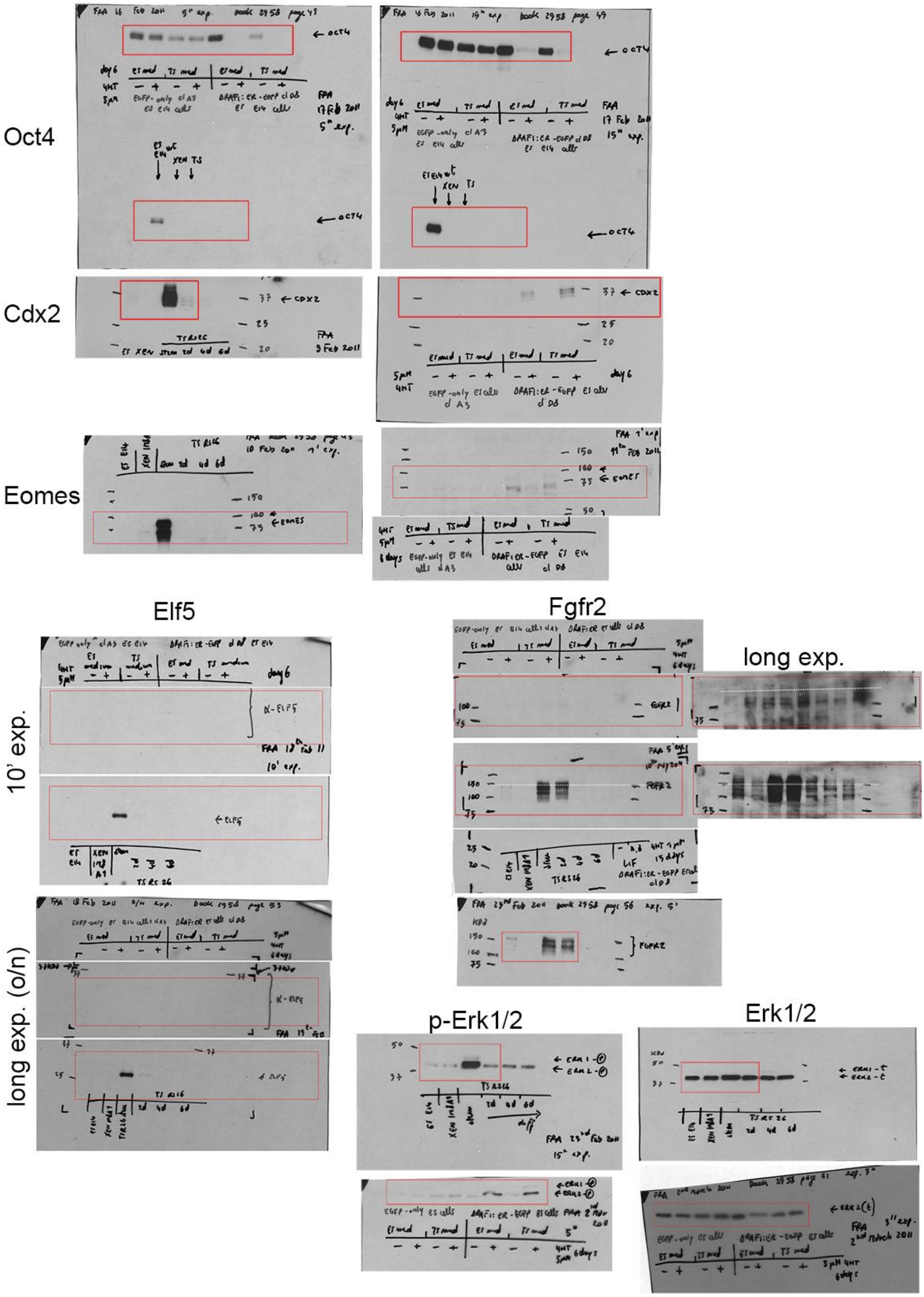

Supplementary Table 1

Antibodies used for Western Blotting

Antibody	WB dilution	Catalogue reference
	Primary antibodies	
mouse anti-Cdx2	1:1000	Biogenex MU392A-UC
goat anti-Elf5	1:1000	Santa Cruz sc-9645
rabbit anti-Eomes	1:1000	Abcam ab23345
mouse anti-Oct4	1:1000	Santa Cruz sc-5279
rabbit anti-Fgfr2	1:1000	Santa Cruz sc-122
rabbit anti-Parp1	1:1000	Cell Signal. 9542
rabbit anti-ER	1:1000	Santa Cruz sc-543
mouse anti-Erk1	1:3000	BD 610031
mouse anti-phospho Erk1/2	1:1000	Cell Signal. 9106
rabbit anti-phospho Frs2α	1:1000	Cell Signal. 3864
rabbit anti-phospho Jnk	1:1000	Cell Signal. 9251
rabbit anti-phospho p38	1:1000	Cell Signal. 9211
mouse anti-GFP	1:1000	Boehringer 1 814 168
rat anti-tubulin	1:2000	Abcam ab6160
	Horseradish peroxidase-conjugated secondary antibodies	
anti-rabbit	1:3000	BioRad 170-6515
anti-rat	1:3000	GE Healthcare NA935
anti-mouse	1:3000	BioRad 170-6516
anti-goat	1:3000	Abcam ab6885

Supplementary Table 2

Primer sequences

Name	Sequence
Ascl2_F	AGCCCGATGGAGCAGGAG
Ascl2_R	CCGAGCAGAGGTCAGTCAGC
Cdkn1c_F	GCGATCCAGACGCAGGAGCC
Cdkn1c_R	TCGCTGTTCTGCTGCGGAGG
Cdx2_F	AGTGAGCTGGCTGCCACACT
Cdx2_R	GCTGCTGCTGCTTCTTCTTGA
Cdx2_UTR Fwd	CTTCAACGTTTGTCCCCAGA
Cdx2_UTR Rev	CACGGAGCTAGGATACATGC
D930020E02(SynB)_F	TCCGGAAAGGGACCTGCCCA
D930020E02(SynB)_R	CAGCAGTAGTGCGGGGTGCC
Elf5 (ORF/UTR)_F	AAGGAAGAAGAATGACAGGATGACG
Elf5 (ORF/UTR)_R	AATCCATCAAATGAGCCTGGTG
Elf5_F	ATTCGCTCGCAAGGTTACTCC
Elf5_R	GGATGCCACAGTTCTCTTCAGG
Eomes_F	TCGCTGTGACGGCCTACCAA
Eomes_R	AGGGGAATCCGTGGGAGATGGA
Esrrb_F	AGTACAAGCGACGGCTGG
Esrrb_R	CCTAGTAGATTCGAGACGATCTTAGTCA
Ezr Fwd	CCACCAACCAGCCAAGAT
Ezr Rev	CCAATCGTCTTTACCACCTGA
Fgf5_Fw	TGTGTCTCAGGGGATTGTAGGAA
Fgf5_Rv	CTGTCTTTTCAGTTCTGTGGATCG
Fgfr2_F	TGCAGCTAGGACGGTAGACA
Fgfr2_R	GTCCAGTACGGTGCTCTCTG
Gata3_F	AGGCAACCACGTCCCGTCCT
Gata3_R	CGGTGTGGTGGCTGCTCAGG
Gata4_Fw	TAGCAGCAGCAGCAGCAGTG
Gata4_Rv	GCATAGCCTTGTGGGGACAG
Gata6 Fw	TCTACACAAGCGACCACCTCAG
Gata6 Rv	GCCAGAGCACACCAAGAATCC
Gcm1_F	GCTCCACAGAGGAAGGCCGC
Gcm1_R	GTTGGTGACCGGGAAGCCGC
Gm52-(SynA)_F	CCTCACCTCCCAGGCCCCTC
Gm52-(SynA)_R	GGCAGGGAGTTTGCCCACGA
Hand1_F	GAACTCAAAAAGACGGATGGTGG
Hand1_R	CGCCCAGACTTGCTGAGG
Hprt1_F	GTCATGCCGACCCGCAGTCC
Hprt1_R	GGCCACAATGTGATGGCCTCC
Id2-F	ACTCGCATCCCACTATCGTC
Id2-R	AATTCAGATGCCTGCAAGGA
Lasp1 Fwd	TGAACTGTCTGGATAAGTACTGGCA
Lasp1 Rev	GGTGAAGGACTGCTTGGGAT
Map3k8 Fwd	CCGACATCTACAGCCTTGGA

Map3k8 Rev	GGTGCCTGCTTGTGGATAAT
Nanog_Fw	TACCTCAGCCTCCAGCAGATG
Nanog_Rv	CCAGATGCGTTCACCAGATAG
Oct4 (Pou5f1)_F	GAAGCCGACAACAATGAGAACC
Oct4 (Pou5f1)_R	CTCCAGACTCCACCTCACACG
Pgk1 Fwd	CTGACTTTGGACAAGCTGGACG
Pgk1 Rev	GCAGCCTTGATCCTTTGGTTG
Plet1 Fwd	CACTATGGCTAACGTCTCTGG
Plet1 Rev	CTGTCGTCCTCCTTCACTG
Prl2c-(Plf)_F	AACGCAGTCCGGAACGGGG
Prl2c-(Plf)_R	TGTCTAGGCAGCTGATCATGCCA
Prl3b1-(Pl2)_F	GCACTCGGGGAACAGCAGCC
Prl3b1-(Pl2)_R	ACTGCCAGCAACAGGAGTGCC
Prl3d1/2/3-(Pl1)_F	TTATCTTGGCCGCAGATGTGT
Prl3d1/2/3-(Pl1)_R	GGAGTATGGATGGAAGCAGTATGAC
Rin3 Fwd	ATTGGTGCTGTGTGTCCACT
Rin3 Rev	GCCTTCCAGGTACAGTATAGCC
Sdha_F	TGGTGAGAACAAGAAGGCATCA
Sdha_R	CGCCTACAACCACAGCATCA
Sh2d3c Fwd	CCACATGAAGAGGCGAAGCA
Sh2d3c Rev	GGATGGGGTAATGAGGTGCTGT
Sox17_Fw	GATACAATGAGCAGCACCTCCAGAC
Sox17_Rv	CTGGTCGTCACTGGCGTATCC
Sox2_Fw	GAGTGGAAACTTTTGTCCGAGA
Sox2_Rv	GAAGCGTGTACTTATCCTTCTTCAT
Tbp_Fw	CGGTCGCGTCATTTTCTCCGC
Tbp_Rv	GTGGGGAGGCCAAGCCCTGA
Tcfap2c_F	GCCGGACGCCATGTTGTGGA
Tcfap2c_R	ACCCCGGTGTGCGAGAGAGG
Tead4 Fwd	TCTAATGCCTTCTTCCTTGTGA
Tead4 Rev	GAGCAGACCTTCGTAGAGCA
Tinagl1 Fwd	TGATTCCAACGACATCTACCA
Tinagl1 Rev	CTTCCATGAGTGCTTGAACAG
Tpbpa_F	ACTGGAGTGCCCAGCACAGC
Tpbpa_R	GCAGTTCAGCATCCAACTGCG
